# Deciphering spatial domains from spatially resolved transcriptomics with Siamese graph autoencoder

**DOI:** 10.1093/gigascience/giae003

**Published:** 2024-02-20

**Authors:** Lei Cao, Chao Yang, Luni Hu, Wenjian Jiang, Yating Ren, Tianyi Xia, Mengyang Xu, Yishuai Ji, Mei Li, Xun Xu, Yuxiang Li, Yong Zhang, Shuangsang Fang

**Affiliations:** BGI Research, Beijing 102601, China; BGI Research, Shenzhen 518083, China; BGI Research, Beijing 102601, China; BGI Research, Shenzhen 518083, China; BGI Research, Beijing 102601, China; BGI Research, Shenzhen 518083, China; BGI Research, Beijing 102601, China; BGI Research, Shenzhen 518083, China; School of Software, Beihang University, Beijing 100191, China; BGI Research, Beijing 102601, China; BGI Research, Shenzhen 518083, China; BGI Research, Shenzhen 518083, China; BGI Research, Qingdao 266555, China; BGI, Tianjin 300308, China; BGI Research, Shenzhen 518083, China; BGI Research, Wuhan 430074, China; BGI Research, Shenzhen 518083, China; BGI Research, Wuhan 430074, China; Guangdong Bigdata Engineering Technology Research Center for Life Sciences, BGI Research, Shenzhen 518083, China; BGI Research, Shenzhen 518083, China; BGI Research, Wuhan 430074, China; Guangdong Bigdata Engineering Technology Research Center for Life Sciences, BGI Research, Shenzhen 518083, China; BGI Research, Beijing 102601, China; BGI Research, Shenzhen 518083, China

**Keywords:** spatial transcriptomics, spatial clustering, graph neural networks

## Abstract

**Background:**

Cell clustering is a pivotal aspect of spatial transcriptomics (ST) data analysis as it forms the foundation for subsequent data mining. Recent advances in spatial domain identification have leveraged graph neural network (GNN) approaches in conjunction with spatial transcriptomics data. However, such GNN-based methods suffer from representation collapse, wherein all spatial spots are projected onto a singular representation. Consequently, the discriminative capability of individual representation feature is limited, leading to suboptimal clustering performance.

**Results:**

To address this issue, we proposed SGAE, a novel framework for spatial domain identification, incorporating the power of the Siamese graph autoencoder. SGAE mitigates the information correlation at both sample and feature levels, thus improving the representation discrimination. We adapted this framework to ST analysis by constructing a graph based on both gene expression and spatial information. SGAE outperformed alternative methods by its effectiveness in capturing spatial patterns and generating high-quality clusters, as evaluated by the Adjusted Rand Index, Normalized Mutual Information, and Fowlkes–Mallows Index. Moreover, the clustering results derived from SGAE can be further utilized in the identification of 3-dimensional (3D) *Drosophila* embryonic structure with enhanced accuracy.

**Conclusions:**

Benchmarking results from various ST datasets generated by diverse platforms demonstrate compelling evidence for the effectiveness of SGAE against other ST clustering methods. Specifically, SGAE exhibits potential for extension and application on multislice 3D reconstruction and tissue structure investigation. The source code and a collection of spatial clustering results can be accessed at https://github.com/STOmics/SGAE/.

## Background

Spatial transcriptomics (ST) represents a newly emerging technology that revolutionizes the comprehensive characterization of tissue organization and architecture [1, 2]. By profiling the spatially resolved gene expression patterns, ST technologies allow scientists to delve into the intricate cellular dynamics within tissues. Based on the underlying methodology, these techniques can be categorized into 2 main categories: (i) imaging-based methods (MERFISH [3] and seqFISH [4]) and (ii) sequencing-based methods (Slide-seq [5] and 10X Visium [6]). As the need for higher-resolution analysis to unravel cellular diversity becomes imperative, advancements such as Stereo-seq [7] have been developed to provide improved resolution over the years. The advent of ST technologies holds immense potential to drive biological discoveries in development, physiology, and a broad range of diseases [8, 9].

In parallel with single-cell RNA sequencing (scRNA-seq) analysis, clustering serves as the initial step in ST data analysis, grouping individual cells based on their gene expression patterns. Similarly, the primary objective for ST data analysis revolves around dissecting tissue into distinct spatial domains. While traditional machine learning approaches have been applied to tackle this task, recent studies have sought to apply deep learning frameworks to learn how to classify spatial spots into specific regions [10–13]. For instance, SpaGCN [12] identifies spatial domains through a graph convolutional network (GCN) framework, while STAGATE [13] deploys a graph attention autoencoder to define spatial clusters. However, such graph neural network–based methods usually suffer from representation collapse, which tends to map spatial spots into the same representation [14]. Consequently, the discriminative capability of spot representation is limited, leading to inaccurate identification of spatial domains.

To tackle the aforementioned challenge, we proposed SGAE, which aims to learn discriminative spot representation and accurately decipher spatial domains. This framework is derived from the dual correlation reduction network [14], which effectively reduces information correlation at the dual level. SGAE adapts this architecture to ST data analysis by constructing a graph that incorporates both gene expression and spatial information. According to benchmarking assessments, SGAE outperforms existing algorithms in the task of domain identification with superior performance. Moreover, SGAE can be extended in the realm of 3-dimensional (3D) tissue structure identification.

## Results

### Overview of SGAE framework

**Figure 1:**
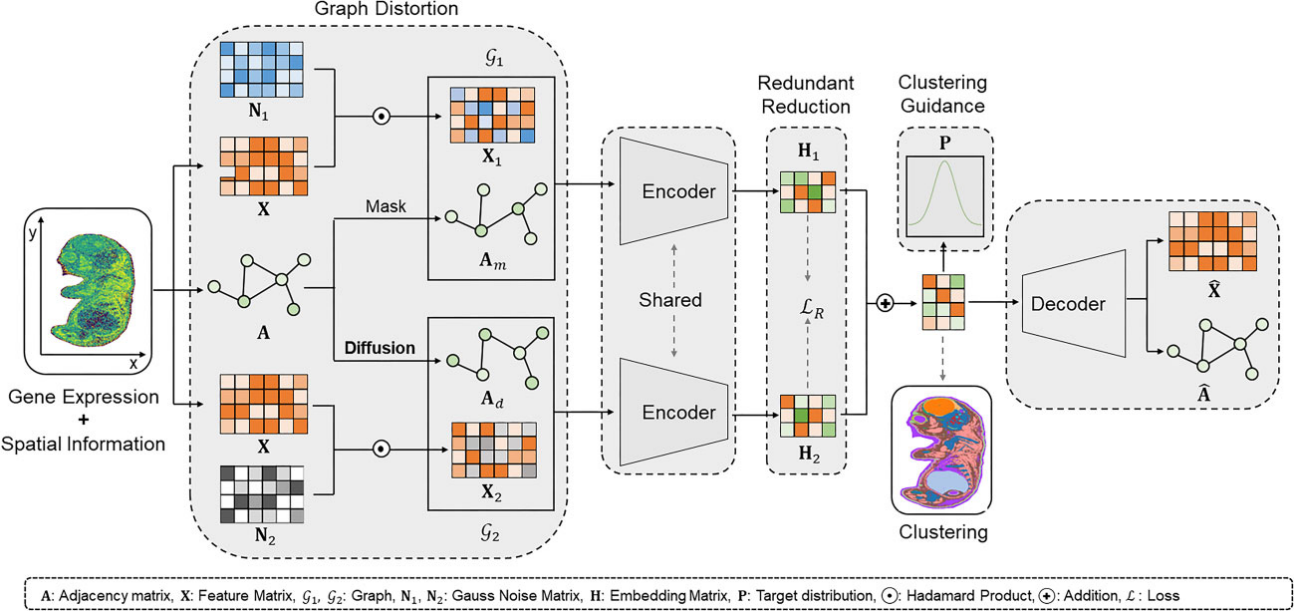
An overview of the SGAE framework. The SGAE algorithm consists of 3 key modules. First, the graph distortion module generates 2 distorted graphs by introducing both attribute and graph disturbances. Second, the encoder module generates 2 sets of representations for each sample. Third, the redundant reduction module ensures that the same sample within the 2 distorted graphs has identical representations at both the feature and sample levels. Last, the discriminative representations are applied to clustering algorithms such as K-means to decipher spatial domains.

SGAE is an unsupervised algorithm for ST clustering that leverages a variational graph autoencoder [15] within a Siamese graph neural network to combine gene expression and spatial information (Fig. 1). To implement SGAE, the gene expression matrix (X) and adjacency matrix (A) are fed into the encoder, which maps the gene expression data into a lower-dimensional latent space, generating embedding vectors (Z) for individual cells. Pseudo-label is first generated by preclustering based on gene expression patterns. SGAE adaptively learns the edge weights of the spatial neighbor network (SNN) to capture the similarity between neighboring spots and update the spot representation by aggregating information from neighbors. Finally, the latent embeddings can be visualized using Uniform Manifold Approximation and Projection (UMAP), and various clustering algorithms such as K-means and Louvain can be employed to identify spatial domains for subsequent analysis.

By calculating K-nearest neighbors based on the relative spatial positioning of spots, SGAE can effectively capture the spatial relationships between cells. This is especially essential for ST data with low spatial resolutions, such as 10X Visium, where discerning fine-grained spatial details can be challenging. Besides, SGAE introduces the concept of a cell type–aware SNN by pruning the SNN based on the preclustering of gene expressions. This preliminary clustering step aids in identifying regions that contain distinct cell types. Through the incorporation of cell-type information during the graph construction process, SGAE adeptly captures data heterogeneity and improves the accuracy of the graph representation.

SGAE uses graph distortion to acquire diverse and informative node representations. This is achieved through the application of 2 types of perturbation: feature perturbation and graph perturbation. For feature perturbation, a random noise matrix is introduced to the feature matrix using the Hadamard product. On the other hand, graph perturbation involves edge removal and graph diffusion within the Siamese architecture. To implement edge removal, a mask matrix is generated based on the cosine similarity matrix computed through pairwise comparisons in the latent space. The 10% of edges with the lowest values are then removed. Graph diffusion is facilitated using a random walk–based Personalized PageRank algorithm [16], allowing for the passage of messages through higher-order neighborhoods. To optimize the learning process, SGAE employs an objective function inspired by the Barlow Twins approach [17], aiming to minimize the deviation of the cross-correlation matrix from the ideal identity matrix and reduce redundant information among nodes in the latent space, therefore improving the overall accuracy of the learned embedding.

### SGAE exhibited remarkable effectiveness and robustness in spatial domain exploration

ST datasets generated by different technology platforms possess distinct resolutions and features, making it essential to validate the clustering robustness of SGAE across these platforms. To achieve this, we included ST datasets generated by 10X Visium, seqFISH [18], MERFISH [3], SLIDE-seq v2 [19], and Stereo-seq [7]. For 10X Visium datasets, samples of human dorsolateral prefrontal cortex were collected, which comprised 12 continuous slides, and each slide has been labeled into 7 layers based on the anatomical structure [20]. For seqFISH, we acquired a sample of mouse gastrulation [21]. In total, 351 genes have been detected and 19,416 cells were labeled into 22 groups. Similar to seqFISH, a mouse primary motor cortex dataset that includes 254 genes and 3,106 cells was detected by MERFISH [22]. As for the SLIDE-seq v2, a mouse olfactory bulb dataset that contains 20,139 cells and 21,220 genes was included to test the performance of SGAE [19]. To test the performance in tissue without a clear structure, the liver cancer dataset from Stereo-seq [23] was utilized. The dataset contains 14,288 spots, and a margin area between cancer and healthy tissue can be seen according to hematoxylin and eosin (H&E) staining. Then we comprehensively compared the clustering performance of SGAE against other state-of-the-art spatial clustering methods, including SpaGCN [12], GraphST [10], STAGATE [13], and Leiden [24]. Clustering performance was assessed by spatial visualization combined with the Adjusted Rand Index (ARI), Normalized Mutual Information (NMI), and Fowlkes–Mallows Index (FMI).

### Human dorsolateral prefrontal cortex 10X Visium dataset

**Figure 2:**
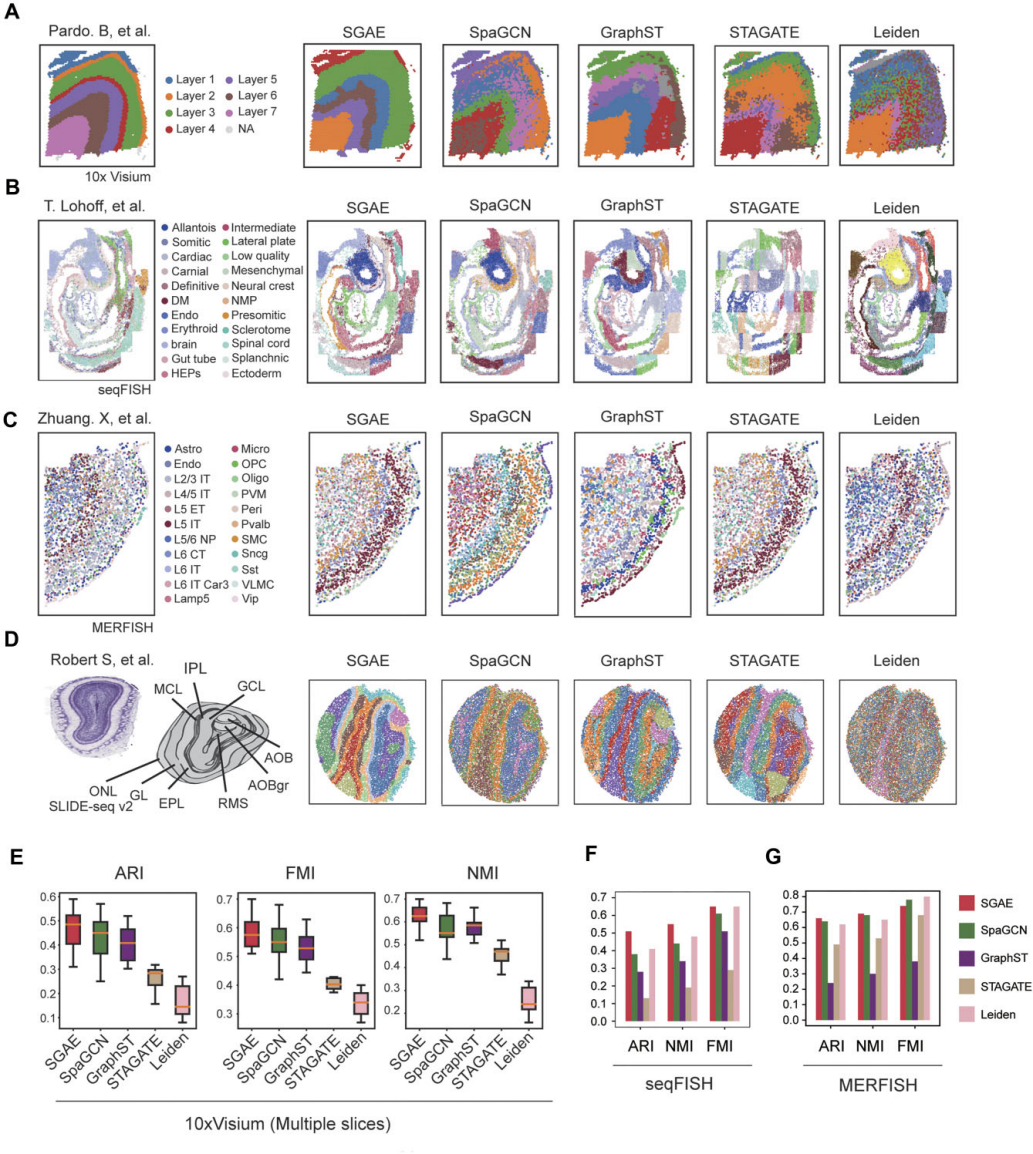
SGAE exhibited high effectiveness and robustness in spatial domain exploration. (A–D) Visualization of clustering results from SGAE, SpaGCN, GraphST, STAGATE, Leiden, and annotation. (A) Human DLPFC 10X Visium dataset. (B) Mouse gastrulation seqFISH dataset. (C) Mouse cortex MERFISH dataset. (D) Mouse olfactory bulb SLIDE-seq v2 dataset. (E–G) Benchmark metrics comparison of SGAE against SpaGCN, GraphST, STAGATE, and Leiden. (E) Boxplot of ARI, FMI, and NMI for 12 DLPFC 10X Visium datasets. (F) Mouse gastrulation seqFISH dataset. (G) Mouse cortex MERFISH dataset.

We applied SGAE to analyze the 10X Visium ST dataset obtained from the human dorsolateral prefrontal cortex (DLPFC) [20]. The visualization of cell clustering confirmed that SGAE was able to discern the intricate stratified cortex structures with remarkable clarity, surpassing the capabilities of other existing methods (Fig. 2A). Furthermore, our benchmarking results revealed that SGAE outperformed other algorithms in analyzing all 12 DLPFC slices (Fig. 2E).

### Mouse gastrulation seqFISH dataset

The evaluation of SGAE’s performance extends to the mouse gastrulation dataset, which was generated through the imaging-based technology seqFISH [21]. The visualization of mouse gastrulation structures derived from different methods demonstrates higher effectiveness of SGAE in accurately discriminating embryo tissue sections (Fig. 2B). In contrast, STAGATE failed to decipher the spatial domain with precision, as it tended to divide the spatial domain into numerous disorder patches. Notably, SGAE reaffirmed its superiority in all benchmark metrics against other methods (Fig. 2F).

### Mouse cortex MERFISH dataset

Based on the MERFISH dataset of the mouse primary motor cortex [22], we further compared the clustering results obtained by different methods. While all 5 methods successfully extracted the stratified structure of the cortex, SGAE demonstrated a remarkable ability to capture the layered organization of the glutamatergic structures more accurately when compared to the original annotation (Fig. 2C). Furthermore, SGAE achieved the highest performance among all 5 methods, underscoring its effectiveness in precisely clustering cells and capturing the spatial arrangement of the primary motor cortex (Fig. 2G).

### Mouse olfactory bulb SLIDE-seq v2 dataset

The evaluation also encompassed the SLIDE-seq V2 dataset of the mouse olfactory bulb [19]. The spatial domains identified by SGAE exhibited remarkable consistency with the annotation provided by the Allen Reference Atlas, strengthening the confidence in its accuracy and reliability (Fig. 2D). Conversely, the Leiden clustering approach failed to provide a cohesive tissue structure in this dataset, while SpaGCN, GraphST, and STAGATE partially deciphered certain structures within the olfactory bulb.

### Liver cancer Stereo-seq dataset

SGAE and alternative clustering methods were tested on a liver cancer sample obtained from Stereo-seq. The application of SGAE resulted in a clearer and more accurate identification of the margin border based on H&E staining (Supplementary Fig. S1A, B). Notably, SGAE also detected clusters consisting of discrete spots located in different positions, reflecting the heterogeneous nature of the tumor tissue. To assess the spatial correlation of the clustering results, we computed Moran’s index. Moran’s index revealed that alternative methods tended to overutilize spatial information and identify clusters in blocks (Supplementary Fig. S1C). To further evaluate the accuracy of the clustering results obtained by these tools, we focused on the rare cell-type fibroblast and used VIM as a marker gene for fibroblasts. We visualized the spatial distribution of VIM and compared it with the most probable cluster identified by each of the methods. The results showed that cluster 6 in SGAE exhibited a higher similarity to the spatial expression of VIM compared to other methods (Supplementary Fig. S1D, E).

Overall, our results unequivocally establish SGAE as a powerful method for analyzing ST data, surpassing other state-of-the-art methods in terms of cell clustering performance and structure exploration of complex tissues.

### SGAE deciphers spatial domains and provides discriminative representations

**Figure 3:**
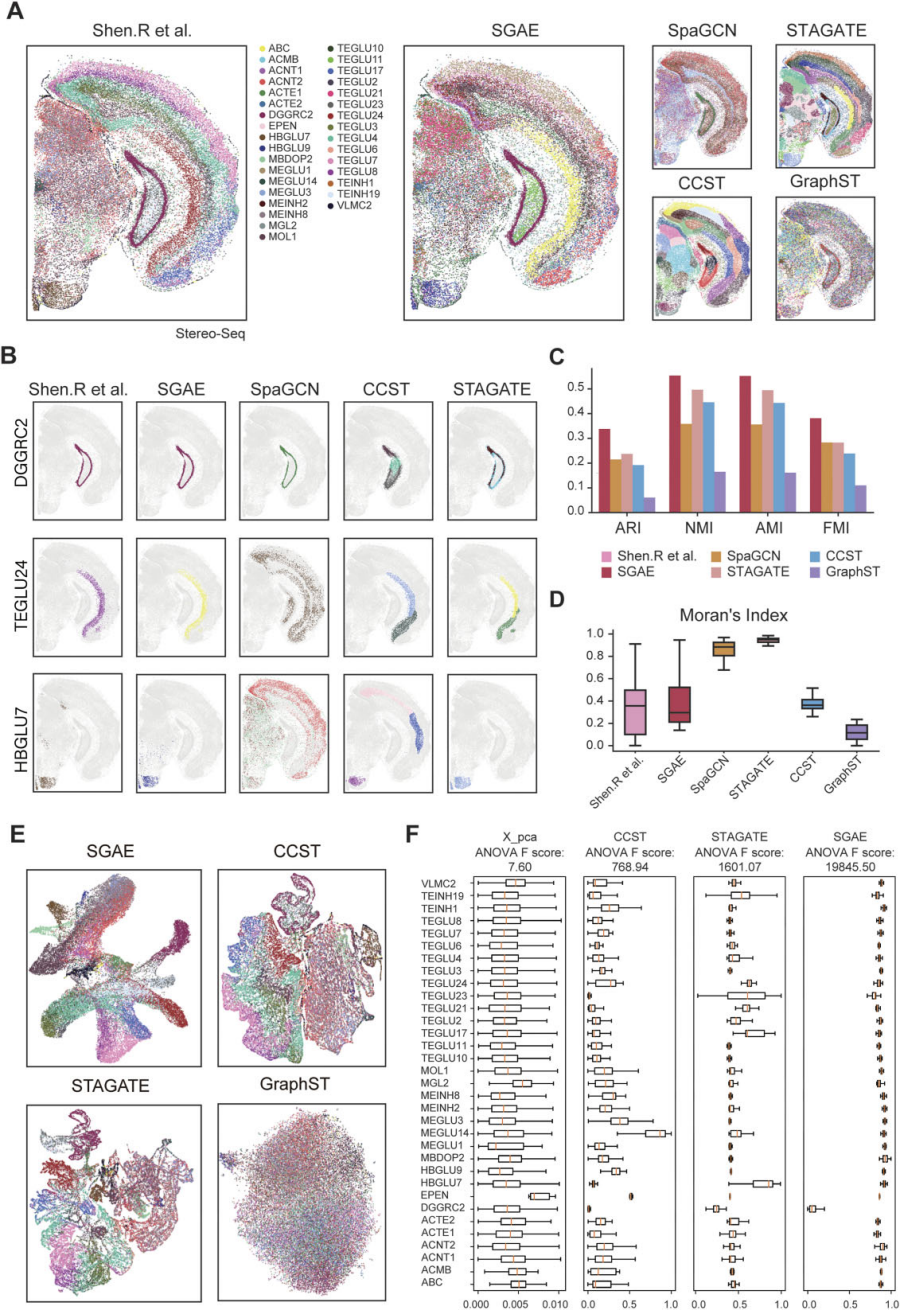
SGAE unraveled spatial domains and provided discriminative representations. (A) Visualization of human adult brain clustering results from SGAE, SpaGCN, STAGATE, CCST, and GraphST. (B) Subclustering results of DGGRC2, TEGLU24, and HBGLU from SGAE, SpaGCN, STAGATE, CCST, and GraphST. (C) Benchmark metrics comparison of SGAE against SpaGCN, STAGATE, CCST, and GraphST. (D) Boxplot of Moran’s index comparison of SGAE against SpaGCN, STAGATE, CCST, and GraphST. (E) UMAP visualization of embedding from SGAE, SpaGCN, STAGATE, and GraphST. (F) Boxplot of ANOVA F-score of pseudo-time calculated from embedding provided by PCA, CCST, STAGATE, and GraphST.

Stereo-seq is a novel ST technology that offers subcellular resolution and has opened up new avenues for investigating the intricate structures within complex tissues [7]. However, the exploitation of its high-resolution capabilities necessitates the utilization of advanced clustering and spatial analysis methods. Therefore, we conducted a meticulous evaluation of SGAE’s clustering performance using a Stereo-seq dataset of the mouse adult brain dataset [25]. It comprises a total of 38,811 cells and 20,062 genes and has been labeled into 38 subclasses through manual annotation. Intriguingly, SGAE showcased exceptional discriminative power in accurately distinguishing mouse brain sections within this dataset, outperforming other methods such as SpaGCN, STAGATE, CCST, and GraphST (Fig. 3A). Subcluster analysis further demonstrated the superior performance of SGAE (Fig. 3B). SGAE accurately delineated distinct subpopulations within the tissue, whereas STAGATE inaccurately divided the DGGRC2 and TEGLU24 regions into 2 separate clusters, and SpaGCN assigned a larger region for TEGLU24 and HBGLU.

To provide a systematic comparison, we conducted an extensive evaluation of SGAE’s clustering results using multiple benchmark metrics, including ARI, NMI, and FMI. Remarkably, SGAE outperformed all other existing methods across all benchmark metrics (Fig. 3C). Besides, we utilized Moran’s index (MI) to assess the spatial autocorrelation of each cluster. Although SpaGCN and STAGATE achieved higher MI scores, SGAE exhibited a distribution most closely aligned with the ground truth in terms of MI (Fig. 3D). It is suggested that SGAE effectively utilizes spatial information in a more reasonable and appropriate manner.

Furthermore, we evaluated the representative embedding provided by SGAE, CCST [11], STAGATE, and GraphST through UMAP visualization (Fig. 3E). The results showed that SGAE exhibited a high level of proficiency in extracting the embedding of the mouse brain Stereo-seq data, while GraphST struggled to distinguish different cell groups. To further evaluate the capability of SGAE to characterize biological representation, we performed pseudo-time analysis and calculated the analysis of variance (ANOVA) F-score for each cell type (Fig. 3F). Surprisingly, SGAE achieved the highest ANOVA F-score, highlighting the discriminative capability of SGAE’s embedding in accurately distinguishing between different cell types.

Taken together, these findings provide compelling evidence that SGAE not only surpasses other methods in terms of clustering accuracy but also excels in providing superior embedding representation for the datasets.

### SGAE enhanced complex spatial domain dissection in 3D *Drosophila*

**Figure 4:**
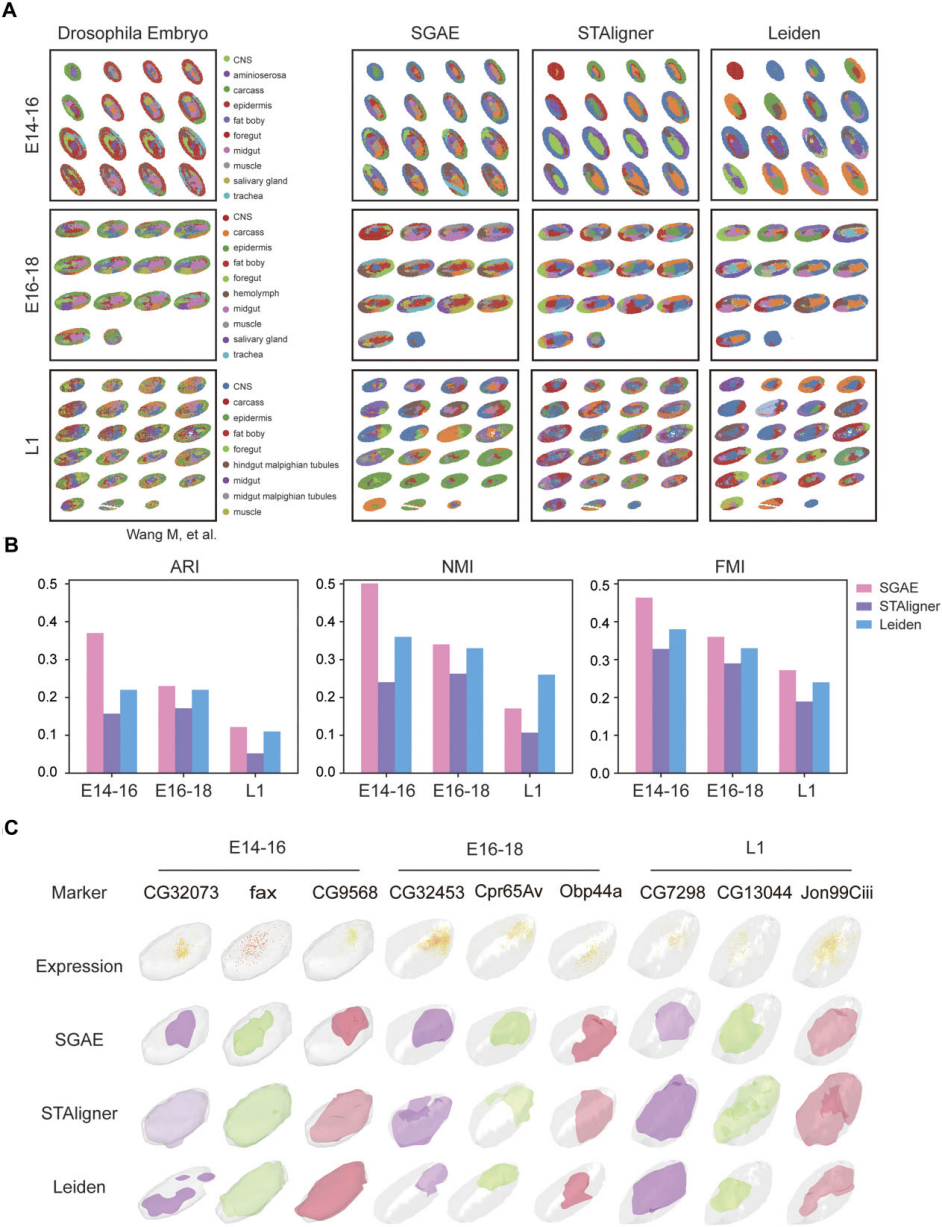
SGAE enhanced complex spatial domain dissection in a 3D *Drosophila* embryo. (A) A 2-dimensional visualization of *Drosophila* embryo clustering results at different stages (E14–16, E16–18, and L1) from SGAE and STAligner. (B) Benchmark metrics comparison of SGAE, Leiden, and STAligner. (C) The 3D visualization of a *Drosophila* embryo. The first row shows the marker genes of the *Drosophila* embryo at different stages, while the last 3 rows display the meshes generated by SGAE, STAligner, and Leiden, respectively.

The advanced use of ST clustering involves integrating 3D reconstruction technology to gain a comprehensive understanding of the spatial organization and gene expression patterns within complex tissues. The fundamental topic of 3D tissue structure dissection is to identify shared and specific spatial domains across multiple slices of ST datasets. Our investigation sought to determine whether SGAE could effectively accomplish this challenging multislice clustering task, especially for the datasets with less batch effect (Supplementary Fig. S2). Notably, we found that SGAE surpassed Leiden and STAligner [26] in accurately dissecting the spatial domains of *Drosophila* embryos at different stages (E14–16, E16–18, and L1) [27], as evidenced by its higher similarity to the ground truth (Fig. 4A, B). These findings highlighted the effectiveness of SGAE in achieving reliable multislice clustering for ST analysis.

After obtaining the clustering results from SGAE, STAligner, and Leiden, we proceeded with the crucial step of stack slice registration to enable 3D tissue reconstruction. This involved aligning consecutive tissue slices to generate a complete and accurate 3D representation of the tissue. We observed that the 3D meshes generated from SGAE results exhibited exceptional accuracy in dividing the tissue into correct structures, aligning perfectly with the corresponding marker genes (Fig. 4C). It indicated that the spatial domains generated by SGAE are highly effective in achieving promising 3D tissue reconstruction. In contrast, STAligner and Leiden faltered in accurately dividing the tissue into correct structures in certain cases. This suggests the robustness and reliability of the spatial domains generated by SGAE.

## Discussion

Spatial transcriptomics is a cutting-edge technology that allows us to simultaneously capture gene expression while retaining spatial information of the tissue. The emergence of large-scale ST data has increased the demand for effective algorithms capable of dissecting spatial domains. To achieve this, we proposed SGAE, a framework composed of 2 identical encoders based on a Siamese network, which enabled us to encode cell features. Additionally, SGAE employs a graph neural network that facilitates the learning of informative representations of both gene expression and spatial locations. To fully leverage the spatial information provided by ST, we constructed a graph based on the spatial information of each cell and preclustered gene expression. We then used a linear combination operation to merge the decorrelated latent embeddings, enhancing the discriminative power of the resulting embedding and clustering accuracy, thus facilitating comprehensive analysis to provide profound insights into complex biological systems.

Our study demonstrates the effectiveness and robustness of SGAE in capturing tissue structures across different ST technology platforms. This superiority over other methods indicates the immense potential of SGAE as a reliable tool for analyzing ST datasets. Another notable strength of SGAE lies in its ability to accurately capture the heterogeneity present within ST datasets. The complexity and diversity of cell types within tissues pose significant challenges in accurately characterizing gene expression patterns. Notably, SGAE’s embedding successfully captures the heterogenic information, enabling a more comprehensive understanding of the spatial organization of gene expression patterns within tissues. While SGAE has demonstrated its advantages in ST clustering, further validation across a wider range of ST datasets and biological systems is necessary to fully assess the generalizability of SGAE’s performance.

In this study, we also applied SGAE to analyze the *Drosophila* 3D dataset and unravel the spatial domains during the E14–16, E16–18, and larva L1 stages. We further compared the performance of SGAE with that of STAligner, a commonly used method developed for multislice ST analysis. By evaluating benchmark metrics, we consistently observed that SGAE outperformed STAligner in effectively grouping cells into biologically meaningful clusters. The superior clustering results of SGAE carry significant implications for the analysis of 3D tissue structure reconstruction. In conclusion, SGAE demonstrates its proficiency in spatial domain identification on spatial transcriptomics with a moderate batch effect. For datasets with a high batch effect, it is recommended to integrate batch removal methods upstream of SGAE to effectively mitigate this issue. By accurately categorizing cells into reasonable groups, SGAE could contribute to a more precise characterization of the spatial organization of gene expression patterns. This is particularly important for understanding the complex processes underlying biological development and differentiation.

## Methods

### Notations and problem definition

An undirected graph is usually represented by ${\mathrm{G}} = \{ {{\mathrm{V}},{\mathrm{\ E}}} \}$, where ${\mathrm{V}} = \{ {{{\mathrm{v}}}_1,{\mathrm{\ }}{{\mathrm{v}}}_2,{\mathrm{\ }} \cdots ,{\mathrm{\ }}{{\mathrm{v}}}_{\mathrm{N}}} \}$ and ${\mathrm{E}}$ are the node and edge, respectively. Each node ${{\mathrm{v}}}_{\mathrm{i}}$ is characterized by a vector ${{\boldsymbol{x}}}_{{\bf i}} \in {{\mathrm{R}}}^{\mathrm{D}}$, where ${\mathrm{D}}$ is the dimension of the attribute. Then the graph can be characterized by the feature matrix ${\mathrm{X}} \in {{\mathrm{R}}}^{{\mathrm{N}} \times {\mathrm{D}}}$. The relation between each node is characterized by the adjacency matrix ${\mathrm{A}} = {( {{{\mathrm{a}}}_{{\mathrm{ij}}}} )}_{{\mathrm{N}} \times {\mathrm{N}}}$, where ${{\mathrm{a}}}_{{\mathrm{ij}}} = 1$ if ${{\mathrm{v}}}_{\mathrm{i}}$ and ${{\mathrm{v}}}_{\mathrm{j}}$ are connected by an edge; otherwise, ${a}_{ij} = 0$. A degree matrix describes the number of edges connected to each node and can be expressed in a diagonal matrix ${\mathrm{D}} = {\mathrm{\ diag}}( {{{\mathrm{d}}}_1,{{\mathrm{d}}}_2,{\mathrm{\ }} \cdots ,{\mathrm{\ }}{{\mathrm{d}}}_{\mathrm{N}}{\mathrm{\ }}} ) \in {{\mathrm{R}}}^{{\mathrm{N}} \times {\mathrm{N}}}$, and ${{\mathrm{d}}}_{\mathrm{i}}$ is the degree of node ${v}_i$ and calculated by ${{\mathrm{d}}}_{\mathrm{i}} = \mathop \sum \limits_{({{\mathrm{v}}}_{\mathrm{i}},{\mathrm{\ }}{{\mathrm{v}}}_{\mathrm{j}}) \in {\mathrm{E}}} {{\mathrm{a}}}_{{\mathrm{ij}}}$. We normalized the adjacency matrix as ${\mathrm{\tilde{A}}} = {D}^{ - 1}( {A\ + \ I} )$, where $I \in {{\mathrm{R}}}^{{\mathrm{N}} \times {\mathrm{N}}}$ is the identity matrix.

In this article, we aimed to train a Siamese graph encoder that embeds all nodes into the low-dimension latent space in an unsupervised manner. The resultant latent embedding can then be directly utilized to perform node clustering by clustering metrics such as K-means and Leiden.

### The overall architecture of SGAE

The overall architecture of SGAE consists of graph distortion, Siamese encoders, Siamese decoders, and a reconstruction loss function.

#### Graph distortion

We utilized 2 types of graph distortion, including feature corruption and edge perturbation.

For feature corruption, which is the feature-level distortion, we applied a Hadamard product to feature matrix and a random noise matrix generated from a Gaussian distribution, that is, $\tilde{X} = X \odot N$, where $ \odot $ means the Hadamard product and $N \sim N( {1,\ 0.1} )$.

For edge perturbation, which is the structure-level distortion, we adopted 2 types of distortion (i.e., edge removal and graph diffusion). For the edge removal, we generated a mask matrix $M$ according to the similarity matrix by calculating the pairwise cosine similarity in the latent space, where 10% of the lowest edges would be removed. The final adjacency matrix after edge removal is


\begin{eqnarray*}
{A}^m = {D}^{ - \frac{1}{2}}\left( {\left( {A \odot M} \right) + I} \right){D}^{ - \frac{1}{2}}
\end{eqnarray*}

In the graph diffusion treatment, we used Personalized PageRank to calculate the normalized adjacency matrix into a graph diffusion matrix by following the MVGRL method [28]:


\begin{eqnarray*}
{A}^d = \alpha {\left( {I - \left( {1 - \alpha } \right)\left( {{D}^{ - \frac{1}{2}}\left( {A + I} \right){D}^{ - \frac{1}{2}}} \right)} \right)}^{ - 1}
\end{eqnarray*}

where $\alpha = 0.2$ as the teleport probability in a random walk.

#### Siamese encoders

In order to reduce the utilization of space while learning richer cell representations, we constructed the 2 same encoders based on the Siamese network structure to encode cell features.

The inputs of the Siamese encoders are graph ${G}_1\ = \ ( {{X}_1,\ {A}_m} )$ and graph ${G}_2\ = \ ( {{X}_2,\ {A}_d} )$. The output is the embedding matrix $H$. First, we used 2 parameter-shared encoders to encode graph ${G}_1$ and graph ${G}_2$, respectively, and generate embedding matrices ${H}_1$ and ${H}_2$. The encoder in the $l$th layer can be formulated as:


\begin{eqnarray*}
{\mathrm{H}}_1^{\left( {\mathrm{l}} \right)} = {\mathrm{\sigma }}\left( {\widehat {{{\mathrm{A}}}_{\mathrm{m}}}{\mathrm{H}}_1^{\left( {{\mathrm{l}} - 1} \right)}{\mathrm{W}}_1^{\left( {\mathrm{l}} \right)}} \right) + {\mathrm{\sigma }}\left( {{\mathrm{H}}_1^{\left( {{\mathrm{l}} - 1} \right)}{\mathrm{W}}_2^{\left( {\mathrm{l}} \right)} + {{\mathrm{b}}}^{\left( {\mathrm{l}} \right)}{\mathrm{\ }}} \right) \end{eqnarray*}

\begin{eqnarray*}
{\mathrm{H}}_2^{\left( {\mathrm{l}} \right)} = {\mathrm{\sigma }}\left( {\widehat {{{\mathrm{A}}}_{\mathrm{d}}}{\mathrm{H}}_2^{\left( {{\mathrm{l}} - 1} \right)}{\mathrm{W}}_1^{\left( {\mathrm{l}} \right)}} \right) + {\mathrm{\sigma }}\left( {{\mathrm{H}}_2^{\left( {{\mathrm{l}} - 1} \right)}{\mathrm{W}}_2^{\left( {\mathrm{l}} \right)} + {{\mathrm{b}}}^{\left( {\mathrm{l}} \right)}{\mathrm{\ }}} \right) \end{eqnarray*}

where $\widehat {{{\mathrm{A}}}_{\mathrm{m}}} = {\mathrm{D}}_{\mathrm{m}}^{ - \frac{1}{2}}( {{{\mathrm{A}}}_{\mathrm{m}} + {\mathrm{I}}} ){\mathrm{D}}_{\mathrm{m}}^{ - \frac{1}{2}},{\mathrm{\ }}\widehat {{{\mathrm{A}}}_{\mathrm{d}}} = {\mathrm{D}}_{\mathrm{d}}^{ - \frac{1}{2}}( {{{\mathrm{A}}}_{\mathrm{d}} + {\mathrm{I}}} ){\mathrm{D}}_{\mathrm{d}}^{ - \frac{1}{2}}$, ${D}_m$ and ${D}_d$ are degree matrices of ${A}_m$ and ${A}_d$, $I$ is the identity matrix, $W_1^{( l )}$ and $W_2^{( l )}$ are weight matrices of encoders in the $l$th layer, ${b}^{( l )}$ is the bias vector of the encoder in the $l$th layer, and $\sigma $ is the nonlinear activate function, such as ReLU and Tanh. When layer $l = 1$, $H_1^{( {l - 1} )} = {X}_1$.

Ultimately, the decorrelated latent embeddings derived from 2 different views—namely, ${H}_1$ and ${H}_2$—are merged using a linear combination operation. This amalgamation produces clustering-focused latent embeddings that can be effectively employed for clustering purposes, particularly through the utilization of the K-means algorithm.

#### Siamese decoders

For SGAE, we constructed a decoder based on graph convolutional neural networks while reconstructing feature embeddings and adjacency matrices. The input is the embedding matrix $H$, and the output is the original feature matrix $X$ and the adjacency matrix $A$. First, we used the graph convolutional neural network to decode the embedding $H$ to generate a feature matrix ${\mathrm{\hat{H}}}$, and the calculation formula of the k layer decoder is as follows:


\begin{eqnarray*}
{{\mathrm{H}}}^{\left( {\mathrm{k}} \right)} = {\mathrm{\sigma }}\left( {{{\mathrm{D}}}^{ - \frac{1}{2}}\left( {{\mathrm{A}} + {\mathrm{I}}} \right){{\mathrm{D}}}^{ - \frac{1}{2}}{{\mathrm{H}}}^{\left( {{\mathrm{k}} - 1} \right)}{{\mathrm{W}}}^{\left( {\mathrm{k}} \right)}} \right) \end{eqnarray*}

where $D$ is the degree matrix of the matrix $A$, and ${W}^{( k )}$ is the parameter matrix of the $k$th layer of the decoder. Then, we took an inner product computation between the embedding matrix $H$ and its transpose to generate the adjacency matrix ${\mathrm{\hat{A}}}$.

#### Reconstruction loss function

Finally, we calculated the feature matrix reconstruction loss ${L}_{REC - F}$ as follows:


\begin{eqnarray*}
{{\mathrm{L}}}_{{\mathrm{REC}} - {\mathrm{F}}} = \frac{1}{{2{\mathrm{N}}}}\left| {\left| {{\mathrm{AX}} - {\mathrm{\hat{H}}}} \right|} \right|_{\mathrm{F}}^2
\end{eqnarray*}

We also calculated the adjacency matrix reconstruction loss ${{\mathrm{L}}}_{{\mathrm{REC}} - {\mathrm{A}}}$ as follows:


\begin{eqnarray*}
{{\mathrm{L}}}_{{\mathrm{REC}} - {\mathrm{A}}} = \frac{1}{{2{\mathrm{N}}}}\left| {\left| {{\mathrm{A}} - {\mathrm{\hat{A}}}} \right|} \right|_{\mathrm{F}}^2
\end{eqnarray*}

The reconstruction loss ${{\mathrm{L}}}_{{\mathrm{REC}}}$ is the sum of the feature matrix reconstruction loss and the adjacency matrix reconstruction loss, and the calculation formula is as follows:


\begin{eqnarray*}
{L}_{REC} = {L}_{REC - F} + {L}_{REC - A}
\end{eqnarray*}

#### Redundant reduction module

In order to eliminate redundant information in node embedding and generate distinguishable embeddings for each node, the present invention designed a de-redundancy module, which eliminated redundant information from 2 levels: node level and feature level:


\begin{eqnarray*}
{{\mathrm{S}}}_{\mathrm{N}} = \frac{{{{\mathrm{H}}}_1{\mathrm{H}}_2^{\mathrm{T}}}}{{||{{\mathrm{H}}}_1\left| {\left| {\mathrm{\ }} \right|\left| {{{\mathrm{H}}}_2} \right|} \right|}}
\end{eqnarray*}

\begin{eqnarray*}
{{\mathrm{S}}}_{\mathrm{F}} = \frac{{{{\mathrm{Z}}}_1{\mathrm{Z}}_2^{\mathrm{T}}}}{{||{{\mathrm{Z}}}_1\left| {\left| {\mathrm{\ }} \right|\left| {{{\mathrm{Z}}}_2} \right|} \right|}}
\end{eqnarray*}

\begin{eqnarray*}
{L}_{RR} = {L}_{RR - N} + {L}_{RR - F}
\end{eqnarray*}

#### Clustering guidance module

In order to effectively learn the feature embedding related to the clustering task, the present invention designed a clustering guidance module. First, we pretrained the model and used K-means to cluster the generated node embeddings. Second, we constructed a clustering guidance loss ${L}_C$ according to the node embedding matrix and the clustering result of the previous step: (i) Compute the soft assignment matrix $Q$ for all nodes and pretrained cluster centers using the Student’s *t* distribution. (ii) Generate the target distribution matrix $P$ according to the soft allocation matrix $Q$, and the element ${p}_{ij}$ of the $i$ row $j$ column is calculated by the following formula:


\begin{eqnarray*}
{{\mathrm{p}}}_{{\mathrm{ij}}} = \frac{{{\mathrm{q}}_{{\mathrm{ij}}}^2/\mathop \sum \nolimits_{\mathrm{i}} {{\mathrm{q}}}_{{\mathrm{ij}}}{\mathrm{\ }}}}{{\mathop \sum \nolimits_{{\mathrm{j^{\prime}}}} \left( {{\mathrm{q}}_{{\mathrm{ij^{\prime}}}}^2/\mathop \sum \nolimits_{\mathrm{i}} {{\mathrm{q}}}_{{\mathrm{ij^{\prime}}}}} \right){\mathrm{\ }}}}
\end{eqnarray*}

Then, we computed the clustering guidance loss ${L}_C$ using the Kullback-Leibler (KL) divergence from the soft assignment, the target distribution, and the pretrained soft assignment.

During training, the model was optimized by minimizing the loss function:


\begin{eqnarray*}
L = {L}_{REC} + {L}_C + {L}_{RR}
\end{eqnarray*}

After the model training was completed, the main flow of data in the model inference process was as follows: first, the model was used to obtain the low-dimensional feature embedding $H$ of cells, and then based on the learned embedding, K-means was used for clustering, and finally the cluster labels of all cells were obtained.

### Clustering refinement

SGAE also incorporates an optional clustering refinement step. During this step, SGAE analyzes the domain assignment of each spot and its neighboring spots. Specifically, for a given spot, the label that appears most frequently among its surrounding spots is assigned to that spot. The clustering refinement step was exclusively performed for the human DLPFC 10X Visium data.

### Performance evaluation

We used 5 indices to evaluate the quality of the clustering results: ARI, NMI, FMI, Adjusted Mutual_Infomation (AMI), and MI. These indices provide different perspectives on the clustering performance. ARI measures the similarity of predicted types in the clusters, with a range from −1 to 1. NMI measures the relationship between variables and is normalized to a range of [0,1]. FMI calculates the geometric mean of pairwise precision and recall, also ranging from 0 to 1. AMI measures the similarity between the cluster assignments obtained from a clustering algorithm and the ground-truth cluster assignments. MI is used to assess spatial autocorrelation in the clustering results. Together, these indices offer a comprehensive evaluation of the clustering quality across various aspects.

Here are formulas and function Application Programming Interfaces (APIs) used to implement the indices.

ARI: sklearn.metrics.adjusted_rand_score


\begin{eqnarray*}
ARI = \frac{{\left( {TP + TN} \right)}}{{C_N^2}}{\mathrm{\ }}\left( {N = \textit{samples}} \right) \end{eqnarray*}

NMI: sklearn.metrics.normalized_mutual_info_score


\begin{eqnarray*}
MI\left( {X,Y} \right) = \mathop \sum \limits_{i = 1}^{\left| X \right|} \mathop \sum \limits_{j = 1}^{\left| Y \right|} P\left( {i,j} \right)\log \left( {\frac{{P\left( {i,j} \right)}}{{P\left( i \right)P\left( j \right)}}} \right) \end{eqnarray*}

\begin{eqnarray*}
H\left( X \right) = {\mathrm{\ }} - \mathop \sum \limits_{i = 1}^{\left| X \right|} P\left( i \right)\log \left( {P\left( i \right)} \right);H\left( Y \right) = {\mathrm{\ }} - \mathop \sum \limits_{j = 1}^{\left| Y \right|} P\left( j \right)\log \left( {P\left( j \right)} \right) \end{eqnarray*}

\begin{eqnarray*}
NMI\left( {X,Y} \right) = {\mathrm{\ }}\frac{{2MI\left( {X,{\mathrm{\ }}Y} \right)}}{{H\left( X \right) + H\left( Y \right)}}
\end{eqnarray*}

FMI: sklearn.metrics.fowlkes_mallows_score


\begin{eqnarray*}
FMI{\mathrm{\ }} = {\mathrm{\ }}TP{\mathrm{\ }}/{\mathrm{\ }}\textit{sqrt}\left( {\left( {TP{\mathrm{\ }} + {\mathrm{\ }}FP} \right){\mathrm{\ *\ }}\left( {TP{\mathrm{\ }} + {\mathrm{\ }}FN} \right)} \right) \end{eqnarray*}

AMI: sklearn.metrics.adjusted_mutual_info_score


\begin{eqnarray*}
AMI\left( {X,Y} \right) = {\mathrm{\ }}\frac{{MI\left( {X,{\mathrm{\ }}Y} \right) - E\left\{ {MI\left( {X,Y} \right)} \right\}}}{{1/2\left( {H\left( X \right) + H\left( Y \right)} \right) - E\left\{ {MI\left( {X,Y} \right)} \right\}}}
\end{eqnarray*}

MI: scanpy.metrics.morans_i


\begin{eqnarray*}
E\left[ I \right] = {\mathrm{\ }} - \frac{1}{{n - 1}};V\left[ I \right] = E\left[ {{I}^2} \right] - E\left[ {{I}^2} \right]{z}_I = \left( {I - E\left[ I \right]} \right)/\sqrt {V\left[ I \right]}
\end{eqnarray*}

\begin{eqnarray*}
{S}_0 = \mathop \sum \limits_{i = 1}^n \mathop \sum \limits_{j = 1}^n {\omega }_{i,j}
\end{eqnarray*}

\begin{eqnarray*}
I = \frac{{n\mathop \sum \nolimits_{i = 1}^n \mathop \sum \nolimits_{j = 1}^n {\omega }_{i,j}{z}_i{z}_j}}{{{S}_0\mathop \sum \nolimits_{i = 1}^n z_i^2}}
\end{eqnarray*}

### Data preprocessing

SGAE utilizes transcriptome-wide gene expression profiles with spatial coordinates as input. The raw gene counts per spot are first normalized to the total counts per cell and then scaled through log-transformation. In the case of 3D *Drosophila* datasets, we did not employ any multislice integration method as there was little batch effect observed from the UMAP result. Principal component analysis was then conducted on the gene expression data using the *sc.pp.pca()* function, and the top 50 principal components per spot were subsequently utilized as the default expression feature.

#### Identifying differentially expressed genes

The Wilcoxon test implemented in SCANPY [29] was used to calculate differentially expressed genes for each spatial domain Benjamin–Hochberg adjustment correlation via *sc.tl.rank_genes_groups()*.

### Spatial trajectory inference

We employed the PAGA algorithm [30] implemented in the SCANPY package to depict spatial trajectory. The PAGA trajectory and PAGA tree were inferred by the *scanpy.tl.paga()* function based on cell embedding generated by SGAE. Furthermore, *scanpy.tl.dpt()* was applied to estimate the pseudo-time as well. To compare the performance of each clustering method with embedding, we calculated trajectory and pseudo-time using methods above with the same parameter settings.

## Availability of Supporting Source Code and Requirements

Project name: SGAE

Project homepage: https://github.com/STOmics/SGAE/

Operating system: Linux

Programming language: Python

License: MIT license

RRID: SCR_024803

## Additional Files

**Supplementary Fig. S1**. SGAE reached good performance on a complex and heterogeneous liver cancer sample. (A) H&E staining of a liver cancer sample. Manually added line indicates the border of tumor and healthy tissue. (B) Clustering result of SGAE and other methods. (C) Moran’s index of the clustering results of SGAE and other methods. (D) Spatial map of the expression of VIM. (E) The most likely clusters associated with fibroblasts identified using SGAE and other methods, determined by the expression of VIM.

**Supplementary Fig. S2**. Less batch effect detected in 3D *Drosophila* embryos. UMAP visualization of 3D *Drosophila* embryos. Left: color in cell type annotation. Right: color in slices of sample. (A) E14–16. (B) E16–18. (C) L1.

## Data Availability

Supporting datasets for this article are available via the following databases: human dorsolateral prefrontal cortex 10X Visium dataset from spatialLIBD [31], mouse cortex MERFISH dataset from Brain Image Library [32], mouse gastrulation seqFISH dataset from SpatialMouseAtlas [21], mouse olfactory bulb SLIDE-seq v2 dataset from Single Cell PORTAL [33], liver cancer Stereo-seq dataset and 3D Drosophila Stereo-seq dataset from CNGBdb [34], and adult mouse brain Stereo-seq dataset from Zenodo [35]. An archival version of SGAE can also be accessed in Software Heritage [36].

## Abbreviations

ANOVA: analysis of variance; ARI: Adjusted Rand Index; DLPFC: dorsolateral prefrontal cortex; FMI: Fowlkes–Mallows Index; GCN: graph convolution network; GNN: graph neural network; H&E: hematoxylin and eosin; MERFISH: multiplexed error-robust fluorescence in situ hybridization; MI: Moran’s index; NMI: Normalized Mutual Information; scRNA-seq: single-cell RNA sequencing; seqFISH: sequential fluorescence in situ hybridization; SNN: spatial neighbor network; ST: spatial transcriptomics; UMAP: Uniform Manifold Approximation and Projection.

## Competing Interests

The authors declare that they have no competing interests.

## Funding

This work is supported by the National Natural Science Foundation for Young Scholars of China(32300526) and National Key R&D Program of China (2022YFC3400400).

## Authors’ Contributions

S.F. and Y.Z. conceived and designed the study. W.J., L.C., C.Y., and Y.R. proposed the SGAE model. L.C., L.H., C.Y., and Y.J. performed the data analysis. T.X. helped with the 3D reconstruction analysis. M.L., X.X, and Y.L. participated in the study discussions. L.C., L.H., C.Y., and S.F. wrote the manuscript.

## Supplementary Material

giae003_SuppFig1

giae003_SuppFig2

giae003_GIGA-D-23-00329_Original_Submission

giae003_GIGA-D-23-00329_Revision_1

giae003_Response_to_Reviewer_Comments_Original_Submission

giae003_Reviewer_1_Report_Original_Submission Jianqi She -- 12/4/2023

giae003_Reviewer_2_Report_Original_Submission Jia Song -- 12/12/2023

giae003_Reviewer_2_Report_Revision_1 Jia Song -- 12/29/2023

giae003_Reviewer_3_Report_Original_Submission Ruoyan Li -- 12/14/2023
