# Supplementary figures and images for: Deciphering spatial domains from spatially resolved transcriptomics with Siamese graph autoencoder
Source: Gigascience. 2024 Feb 20;13:giae003. doi: 10.1093/gigascience/giae003 (PMC10939418; doi:10.1093/gigascience/giae003)

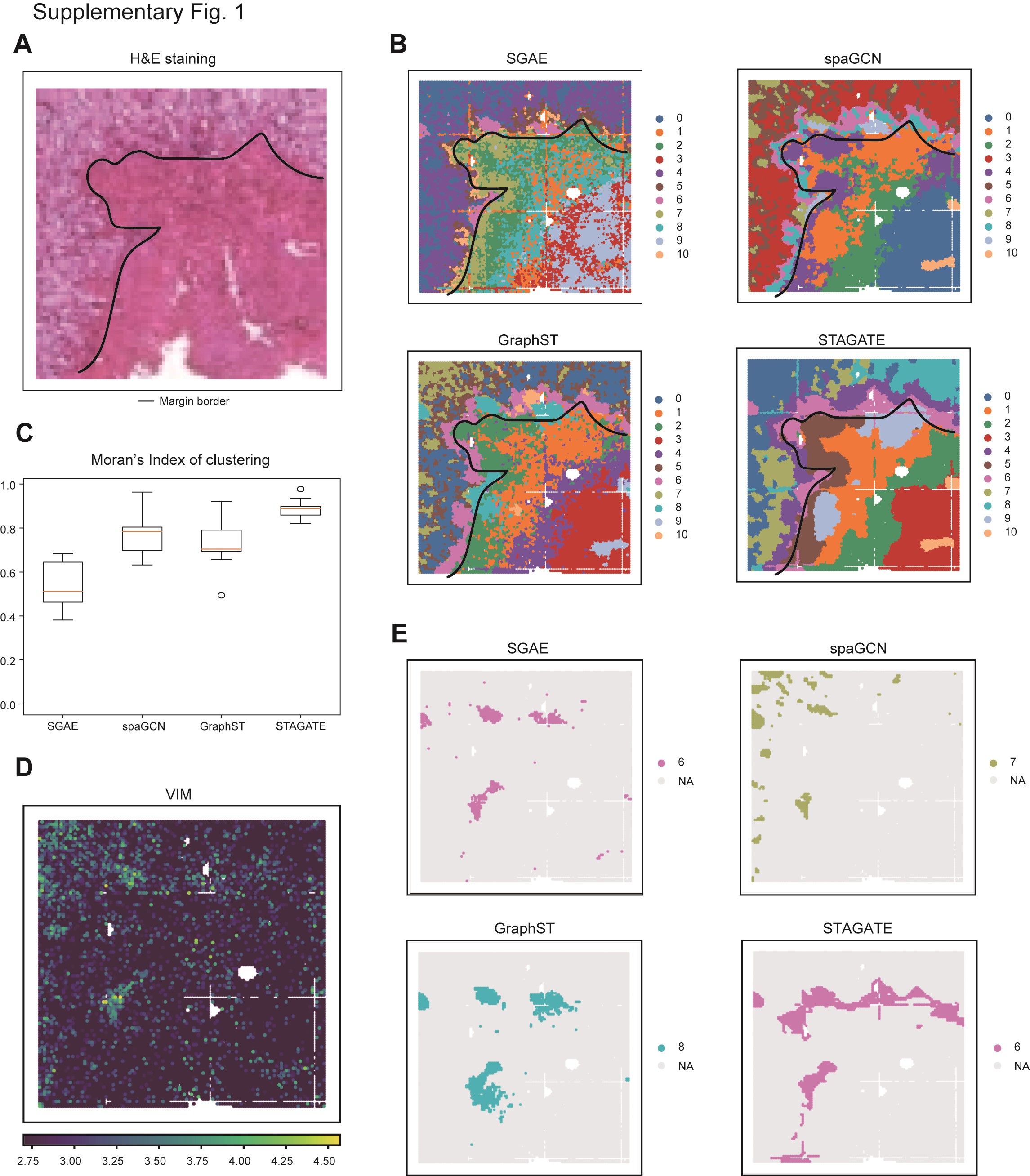

Supplement: giae003_SuppFig1 [file giae003_suppfig1.jpeg]

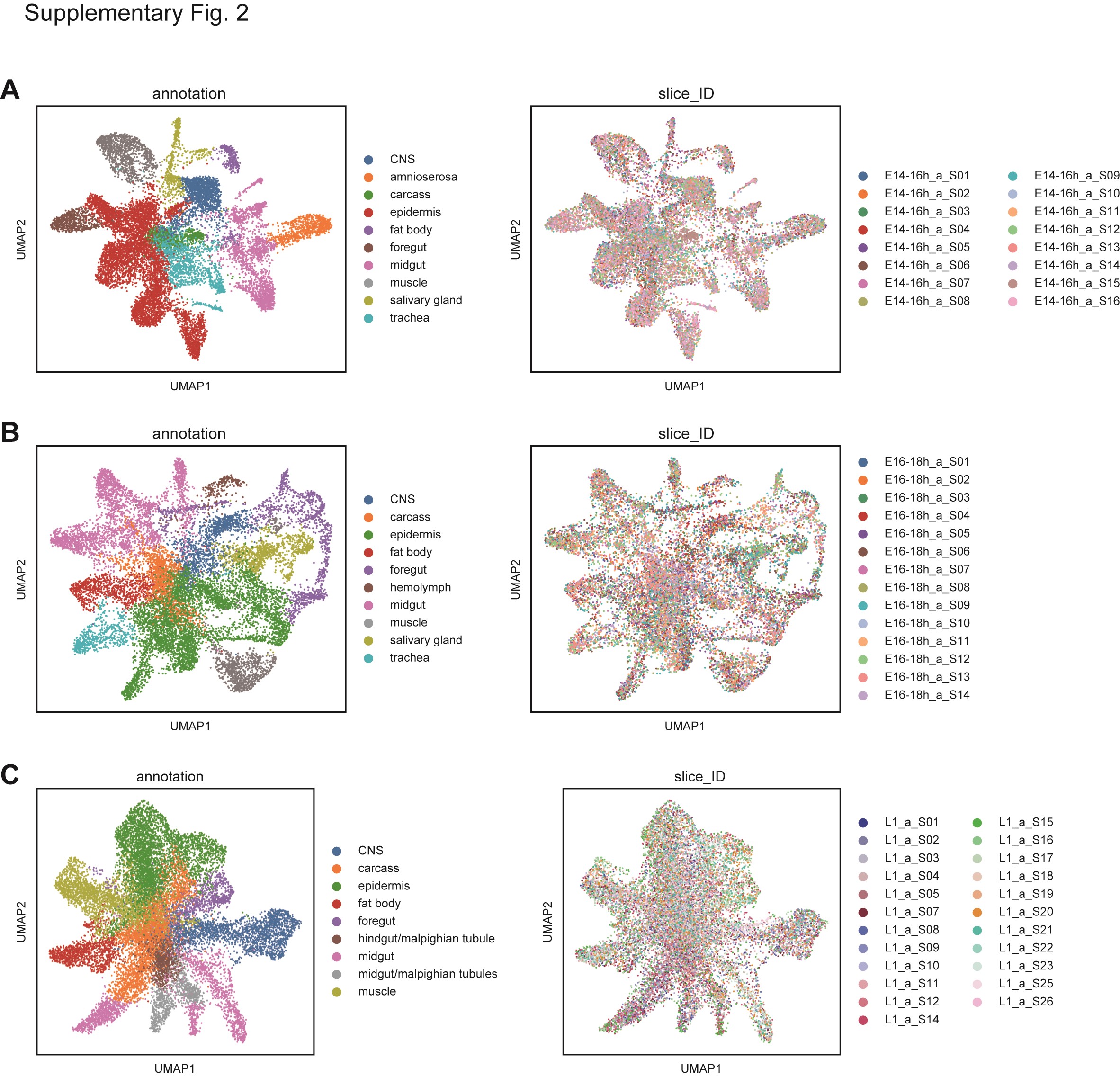

Supplement: giae003_SuppFig2 [file giae003_suppfig2.jpeg]
