# Supplementary material for: Deciphering spatial domains from spatially resolved transcriptomics with Siamese graph autoencoder
Source: Gigascience. 2024 Feb 20;13:giae003. doi: 10.1093/gigascience/giae003 (PMC10939418; doi:10.1093/gigascience/giae003)
Supplement: giae003_GIGA-D-23-00329_Revision_1 [file giae003_giga-d-23-00329_revision_1.pdf]

GigaScience

Deciphering spatial domains from spatially resolved transcriptomics with Siamese Graph Autoencoder

--Manuscript Draft--

**Manuscript Number:**	GIGA-D-23-00329R1
**Full Title:**	Deciphering spatial domains from spatially resolved transcriptomics with Siamese Graph Autoencoder
**Article Type:**	Technical Note
**Funding Information:**	National Natural Science Foundation of China (32300526) — Dr. Shuangsang Fang<br>National Key R&D Program of China (2022YFC3400400) — Pro. Xun Xu

Abstract:

Background

Cell clustering is a pivotal aspect of spatial transcriptomics (ST) data analysis as it forms the foundation for subsequent data mining. Recent advances in spatial domain identification have leveraged Graph Neural Network approaches in conjunction with spatial transcriptomics data. However, such GNN-based methods suffer from representation collapse, wherein all spatial spots are projected onto a singular representation. Consequently, the discriminative capability of individual representation feature is limited, leading to suboptimal clustering performance.

Results

To address this issue, we proposed SGAE, a novel framework for spatial domain identification, incorporating the power of Siamese Graph Autoencoder. SGAE mitigates the information correlation at the both sample and feature level, thus improving the representation discrimination. We adapted this framework to ST analysis by constructing a graph based on both gene expression and spatial information. SGAE outperformed alternative methods by its effectiveness in capturing spatial patterns and generating high-quality clusters, as evaluated by ARI, FMI, and NMI. Moreover, the clustering results derived from SGAE can be further utilized in the identification of 3D Drosophila embryonic structure with enhanced accuracy.

Conclusions

Benchmarking results from various ST datasets generated by diverse platforms demonstrate compelling evidence for the effectiveness of SGAE against other ST clustering methods. Specifically, SGAE exhibits potential for extension and application on multi-slice 3D reconstruction and tissue structure investigation. The source code and a collection of spatial clustering results can be accessed at https://github.com/STOmics/SGAE/.

**Corresponding Author:**	Shuangsang Fang<br>BGI Group<br>BeiJing, CHINA
**Corresponding Author Secondary Information:**	
**Corresponding Author's Institution:**	BGI Group
**Corresponding Author's Secondary Institution:**	
**First Author:**	Shuangsang Fang
**First Author Secondary Information:**	
**Order of Authors:**	Shuangsang Fang

Powered by Editorial Manager® and ProduXion Manager® from Aries Systems Corporation

	Lei Cao
	Chao Yang
	Luni Hu
	Wenjian Jiang
	Yating Ren
	Tianyi Xia
	Mengyang Xu
	Yishuai Ji
	Mei Li
	Xun Xu
	Yuxiang Li
	Yong Zhang
**Order of Authors Secondary Information:**	
**Response to Reviewers:**	Dear Editor,<br><br>Thank you for handling our manuscript entitled "Deciphering spatial domains from spatially resolved transcriptomics with Siamese Graph Autoencoder" and providing us with an opportunity to revise our work. We are delighted to receive positive remarks from both the editors and reviewers regarding our study. We would also like to express our sincere appreciation for the constructive comments provided by both the editors and reviewers, which have greatly strengthened our work.<br><br>We are grateful to the editors for sharing the comments of all three reviewers with us. In this revision, we have diligently addressed all the suggested experiments proposed by reviewers. Please find our point-by-point responses to the editors' comments in the cover letter.<br><br>Sincerely,<br>Shuangsang Fang<br>fangshuangsang@genomics.cn
**Additional Information:**	
**Question**	**Response**
Are you submitting this manuscript to a special series or article collection?	No
**Experimental design and statistics**<br><br>Full details of the experimental design and statistical methods used should be given in the Methods section, as detailed in our Minimum Standards Reporting Checklist. Information essential to interpreting the data presented should be made available in the figure legends.<br><br>Have you included all the information requested in your manuscript?	Yes

**Resources**<br><br>A description of all resources used, including antibodies, cell lines, animals and software tools, with enough information to allow them to be uniquely identified, should be included in the Methods section. Authors are strongly encouraged to cite Research Resource Identifiers (RRIDs) for antibodies, model organisms and tools, where possible.<br><br>Have you included the information requested as detailed in our Minimum Standards Reporting Checklist?	Yes
**Availability of data and materials**<br><br>All datasets and code on which the conclusions of the paper rely must be either included in your submission or deposited in publicly available repositories (where available and ethically appropriate), referencing such data using a unique identifier in the references and in the “Availability of Data and Materials” section of your manuscript.<br><br>Have you have met the above requirement as detailed in our Minimum Standards Reporting Checklist?	Yes

Powered by Editorial Manager® and ProduXion Manager® from Aries Systems Corporation

Manuscript

Click here to access/download;Manuscript;SGAE_final_final.docx

Deciphering spatial domains from spatially resolved transcriptomics with Siamese Graph Autoencoder

Lei Cao[1, 2 †], Chao Yang[1, 2 †], Luni Hu[1, 2 †], Wenjian Jiang[1, 2, ‡], Yating Ren[3], Tianyi Xia[1, 2], Mengyang Xu[2,4], Yishuai Ji[5], Mei Li[2], Xun Xu[6], Yuxiang Li[2, 6, 7], Yong Zhang[2, 6, 7 *], Shuangsang Fang[1, 2 *]

1 BGI Research, Beijing 102601, China.

2 BGI Research, Shenzhen 518083, China.

3 School of Software, Beihang University, Beijing, 100191, China

4 BGI Research, Qingdao 266555, China.

5 BGI, Tianjin 300308, China.

6 BGI Research, Wuhan 430074, China.

7 Guangdong Bigdata Engineering Technology Research Center for Life Sciences, BGI research, Shenzhen 518083, China.

* Corresponding: fangshuangsang@genomics.cn, zhangyong2@stomics.tech

† These authors contributed equally as the first authors.

‡ Senior author.

ORCID iDs: Shuangsang Fang [0000-0002-4126-0074]; Lei Cao [0000-0002-7170-9602]; Chao Yang; Luni Hu; Wenjian Jiang; Yating Ren; Tianyi Xia; Mengyang Xu [0000-0002-4487-7088]; Yishuai Ji; Mei Li; Xun Xu [0000-0002-5338-5173]; Yuxiang Li [0000-0002-1575-3692]; Yong Zhang [0000-0001-9950-1793];

Abstract

Background

Cell clustering is a pivotal aspect of spatial transcriptomics (ST) data analysis as it forms the foundation for subsequent data mining. Recent advances in spatial domain identification have leveraged Graph Neural Network approaches in conjunction with spatial transcriptomics data. However, such GNN-based methods suffer from representation collapse, wherein all spatial spots are projected onto a singular representation. Consequently, the discriminative capability of individual representation feature is limited, leading to suboptimal clustering performance.

Results

To address this issue, we proposed SGAE, a novel framework for spatial domain identification, incorporating the power of Siamese Graph Autoencoder. SGAE mitigates the information correlation at both sample and feature level, thus improving the representation discrimination. We adapted this framework to ST analysis by constructing a graph based on both gene expression and spatial information. SGAE outperformed alternative methods by its effectiveness in capturing spatial patterns and generating high-quality clusters, as evaluated by ARI, FMI, and NMI. Moreover, the clustering results derived from SGAE can be further utilized in the identification of 3D Drosophila embryonic structure with enhanced accuracy.

Conclusions

Benchmarking results from various ST datasets generated by diverse platforms demonstrate compelling evidence for the effectiveness of SGAE against other ST clustering methods. Specifically, SGAE exhibits potential for extension and application on multi-slice 3D reconstruction and tissue structure investigation. The source code and a collection of spatial clustering results can be accessed at https://github.com/STOmics/SGAE/.

Keywords: Spatial transcriptomics; Spatial clustering; Graph neural networks

Background

Spatial transcriptomics (ST) represents a newly emerging technology that revolutionizes the comprehensive characterization of tissue organization and architecture[1, 2]. By profiling the spatially-resolved gene expression patterns, ST technologies allow scientists to delve into the intricate cellular dynamics within tissues. Based on the underlying methodology, these techniques can be categorized into two main categories: (1) imaging-based methods (MERFISH[3] and seqFISH[4]), and (2) sequencing-based methods (Slide-seq[5] and 10x Visium[6]). As the need for higher-resolution analysis to unravel cellular diversity becomes imperative, advancements such as Stereo-seq[7] have been developed to provide improved resolution over the years. The advent of ST technologies holds immense potential to drive biological discoveries in development, physiology and a broad range of diseases[8, 9].

In parallel with single-cell RNA sequencing (scRNA-Seq) analysis, clustering serves as the initial step in ST data analysis, grouping individual cells based on their gene expression patterns. Similarly, the primary objective for ST data analysis revolves around dissecting tissue into distinct spatial domains. While traditional machine learning approaches have been applied to tackle this task, recent studies have sought to apply deep learning frameworks to learn how to classify spatial spots into specific regions[10-13]. For instance, SpaGCN[12] identifies spatial domains through a graph convolutional network (GCN) framework, while STAGATE[13] deploys a graph attention autoencoder to define spatial clusters. However, such graph neural network (GNN) based methods usually suffer from representations collapse, which tends to map spatial spots into the same representation[14]. Consequently, the discriminative capability of spot representation is limited, leading to inaccurate identification of spatial domains.

To tackle the aforementioned challenge, we proposed SGAE, which aims to learn discriminative spot representation and accurately decipher spatial domains. This framework is derived from the Dual Correlation Reduction Network (DCRN)[14], which effectively reduces information correlation at the dual level. SGAE adapts this architecture to ST data analysis by constructing a graph that incorporates both gene expression and spatial information. According to benchmarking assessments, SGAE outperforms existing algorithms in the task of domain identification with

superior performance. Moreover, SGAE can be extended in the realm of 3D tissue structure identification.

Results

Overview of SGAE framework

SGAE is an unsupervised algorithm for ST clustering that leverages a Variational Graph Autoencoder (VGAE)[15] within a Siamese graph neural network to combine gene expression and spatial information (Fig.1). To implement SGAE, the gene expression matrix (X) and adjacency matrix (A) are fed into the encoder, which maps the gene expression data into a lower-dimensional latent space, generating embedding vectors (Z) for individual cells. Pseudo-label is firstly generated by pre-clustering based on gene expression patterns. SGAE adaptively learns the edge weights of the spatial neighbor network (SNN) to capture the similarity between neighboring spots and update the spot representation by aggregating information from neighbors. Finally, the latent embeddings can be visualized using Uniform Manifold Approximation and Projection (UMAP) and various clustering algorithms such as K-means and Louvain can be employed to identify spatial domains for subsequent analysis.

By calculating K-nearest neighbors (KNN) based on the relative spatial positioning of spots, SGAE can effectively capture the spatial relationships between cells. This is especially essential for spatial transcriptomics (ST) data with low spatial resolutions, such as 10x Visium, where discerning fine-grained spatial details can be challenging. Besides, SGAE introduces the concept of a cell type-aware SNN by pruning the SNN based on the pre-clustering of gene expressions. This preliminary clustering step aids in identifying regions that contain distinct cell types. Through the incorporation of cell type information during the graph construction process, SGAE adeptly captures data heterogeneity and improve the accuracy of the graph representation.

SGAE uses graph distortion to acquire diverse and informative node representations. This is achieved through the application of two types of perturbation: feature perturbation and graph perturbation. For feature perturbation, a random noise matrix is introduced to the feature matrix using the Hadamard product. On the other hand, graph perturbation involves edge removal and

graph diffusion within the Siamese architecture. To implement edge removal, a mask matrix is generated based on the cosine similarity matrix computed through pairwise comparisons in the latent space. The 10% of edges with the lowest values are then removed. Graph diffusion is facilitated using a random walk-based Personalized PageRank algorithm[16], allowing for the passage of messages through higher-order neighborhoods. To optimize the learning process, SGAE employs an objective function inspired by the Barlow Twins approach[17], aiming to minimize the deviation of the cross-correlation matrix from the ideal identity matrix and reduce redundant information among nodes in the latent space, therefore improving the overall accuracy of the learned embedding.

SGAE exhibited remarkable effectiveness and robustness in spatial domain exploration

ST datasets generated by different technology platforms possess distinct resolutions and features, making it essential to validate the clustering robustness of SGAE across these platforms. To achieve this, we included ST datasets generated by 10x Visium, seqFISH [18], MERFISH [3], SLIDE-seq v2 [19], and Stereo-seq [7]. For 10x Visium datasets, samples of human dorsolateral prefrontal cortex have been collected, which comprises 12 continuous slides, and each slide has been labeled into 7 layers based on the anatomical structure [20]. For seqFISH, we acquired a sample of mouse gastrulation [21]. 351 genes have been detected and 19416 cells were labeled into 22 groups. Similar to seqFISH, a mouse primary motor cortex dataset which includes 254 genes and 3106 cells was detected by MERFISH [22]. As for the SLIDE-seq v2, a mouse olfactory bulb dataset which contains 20139 cells and 21220 genes was included to test the performance of SGAE [19]. To test the performance in tissue without clear structure. Liver cancer from Stereo-seq [23] is utilized. The dataset contains 14288 spots and a margin area between cancer and healthy tissue can be seen according to the H&E staining. Then we comprehensively compare the clustering performance of SGAE against other state-of-the-art spatial clustering methods, including SpaGCN[12], GraphST[10], STAGATE[13] and Leiden[24]. Clustering performance was assessed by spatial visualization combined with Adjusted Rand Index (ARI), Normalized Mutual Information (NMI) and Fowlkes-Mallows Index (FMI).

Human dorsolateral prefrontal cortex 10x Visium dataset

We applied SGAE to analyze the 10x Visium ST dataset obtained from the human dorsolateral prefrontal cortex (DLPFC)[20]. The visualization of cell clustering confirmed that SGAE was able to discern the intricate stratified cortex structures with remarkable clarity, surpassing the capabilities of other existing methods (Fig. 2A). Furthermore, our benchmarking results revealed that SGAE outperformed other algorithms in analyzing all 12 DLPFC slices (Fig. 2E).

Mouse gastrulation seqFISH dataset

The evaluation of SGAE's performance extends to the mouse gastrulation dataset, which was generated through the imaging-based technology seqFISH[21]. The visualization of mouse gastrulation structures derived from different methods demonstrates higher effectiveness of SGAE in accurately discriminating embryo tissue sections (Fig. 2B). In contrast, STAGATE failed to decipher the spatial domain with precision, as it tends to divide the spatial domain into numerous disorder patches. Notably, SGAE reaffirmed its superiority in all benchmark metrics against other methods (Fig. 2F).

Mouse cortex MERFISH dataset

Based on the MERFISH dataset of the mouse primary motor cortex[22], we further compare the clustering results obtained by different methods. While all five methods successfully extract the stratified structure of the cortex, SGAE demonstrates a remarkable ability to capture the layered organization of the glutamatergic structures more accurately when compared to the original annotation (Fig. 2C). Furthermore, SGAE achieved the highest performance among all five methods, underscoring its effectiveness in precisely clustering cells and capturing the spatial arrangement of the primary motor cortex (Fig 2G).

Mouse olfactory bulb SLIDE-seq v2 dataset

The evaluation also encompasses the SLIDE-seq V2 dataset of the mouse olfactory bulb[19]. The spatial domains identified by SGAE exhibited remarkable consistency with the annotation provided by the Allen Reference Atlas, strengthening the confidence in its accuracy and reliability (Fig. 2D). Conversely, the Leiden clustering approach failed to provide a cohesive tissue structure

in this dataset, while SpaGCN, GraphST, and STAGATE partially deciphered certain structures within the olfactory bulb.

Liver cancer Stereo-seq dataset

SGAE and alternative clustering methods were tested on a liver cancer sample obtained from Stereo-seq. The application of SGAE resulted in a clearer and more accurate identification of the margin border based on H&E staining (Supplementary Fig 1A-B). Notably, SGAE also detected clusters consisting of discrete spots located in different positions, reflecting the heterogeneous nature of the tumor tissue. To assess the spatial correlation of the clustering results, we computed the Moran's Index. The Moran's Index revealed that alternative methods tended to overutilize spatial information and identify clusters in blocks (Supplementary Fig 1C). To further evaluate the accuracy of the clustering results obtained by these tools, we focused on the rare cell type fibroblast and used VIM as a marker gene for fibroblasts. We visualized the spatial distribution of VIM and compared it with the most probable cluster identified by each of the methods. The results showed that Cluster 6 in SGAE exhibited a higher similarity to the spatial expression of VIM compared to other methods (Supplementary Fig 1D-E).

Overall, our results unequivocally establish SGAE as a powerful method for analyzing ST data, surpassing other state-of-the-art methods in terms of cell clustering performance and structure exploration of complex tissues.

SGAE deciphers spatial domains and provides discriminative representations

Stereo-seq is a novel ST technology that offers subcellular resolution and has opened up new avenues for investigating the intricate structures within complex tissues[7]. However, the exploitation of its high-resolution capabilities necessitates the utilization of advanced clustering and spatial analysis methods. Therefore, we conducted a meticulous evaluation of SGAE's clustering performance using a Stereo-seq dataset of the mouse adult brain dataset [25]. It comprises a total of 38811 cells and 20062 genes and has been labeled into 38 subclasses through manually annotation. Intriguingly, SGAE showcased exceptional discriminative power in accurately distinguishing mouse brain sections within this dataset, outperforming other methods

such as SpaGCN, STAGATE, CCST, and GraphST (Fig. 3A). Subcluster analysis further demonstrated the superior performance of SGAE (Fig. 3B). SGAE accurately delineated distinct subpopulations within the tissue, whereas STAGATE inaccurately divided the DGGRC2 and TEGLU24 regions into two separate clusters, and SpaGCN assigned a larger region for TEGLU24 and HBGLU.

To provide a systematic comparison, we conducted an extensive evaluation of SGAE's clustering results using multiple benchmark metrics, including ARI, NMI, and FMI. Remarkably, SGAE outperformed all other existing methods across all benchmark metrics (Fig. 3C). Besides, we utilized Moran's Index (MI) to assess the spatial autocorrelation of each cluster. Although SpaGCN and STAGATE achieved higher MI scores, SGAE exhibited a distribution most closely aligned with the ground truth in terms of MI (Fig. 3D). It is suggested that SGAE effectively utilizes spatial information in a more reasonable and appropriate manner.

Furthermore, we evaluated the representative embedding provided by SGAE, CCST[11], STAGATE, and GraphST through UMAP visualization (Fig. 3E). The results showed that SGAE exhibited a high-level of proficiency in extracting the embedding of the mouse brain Stereo-seq data, while GraphST struggled to distinguish different cell groups. To further evaluate the capability of SGAE to characterize biological representation, we performed pseudotime analysis and calculated the ANOVA F-score for each cell type (Fig. 3F). Surprisingly, SGAE achieved the highest ANOVA F-score, highlighting the discriminative capability of SGAE's embedding in accurately distinguishing between different cell types.

Taken together, these findings provide compelling evidence that SGAE not only surpasses other methods in terms of clustering accuracy, but also excels in providing superior embedding representation for the datasets.

SGAE enhanced complex spatial domain dissection in 3D Drosophila

The advanced use of ST clustering involves integrating 3D reconstruction technology to gain a comprehensive understanding of the spatial organization and gene expression patterns within

complex tissues. The fundamental topic of 3D tissue structure dissection is to identify shared and specific spatial domains across multiple slices of ST datasets. Our investigation sought to determine whether SGAE could effectively accomplish this challenging multi-slice clustering task, especially for the datasets with less batch effect (Supplementary Figure 2). Notably, we found that SGAE surpassed Leiden and STAligner[26] in accurately dissecting the spatial domains of Drosophila embryos at different stages (E14-16, E16-18 and L1)[27], as evidenced by its higher similarity to the ground truth (Fig4. A, B). These findings highlighted the effectiveness of SGAE in achieving reliable multi-slice clustering for ST analysis.

After obtaining the clustering results from SGAE, STAligner and Leiden, we proceeded with the crucial step of stack slice registration to enable 3D tissue reconstruction. This involved aligning consecutive tissue slices to generate a complete and accurate 3D representation of the tissue. We observed that the 3D meshes generated from SGAE results exhibited exceptional accuracy in dividing the tissue into correct structures, aligning perfectly with the corresponding marker genes (Fig. 4C). It indicated that the spatial domains generated by SGAE are highly effective in achieving promising 3D tissue reconstruction. In contrast, STAligner and Leiden faltered in accurately dividing the tissue into correct structures in certain cases. This suggests the robustness and reliability of the spatial domains generated by SGAE.

Discussion

Spatial transcriptomics is a cutting-edge technology that allows us to simultaneously capture gene expression while retain spatial information of the tissue. The emergence of large-scale ST data has increased the demand for effective algorithms capable of dissecting spatial domains. To achieve this, we proposed SGAE, a framework composed of two identical encoders based on a Siamese network, which enabled us to encode cell features. Additionally, SGAE employs a graph neural network that facilitates the learning of informative representations of both gene expression and spatial locations. To fully leverage the spatial information provided by ST, we constructed a graph based on the spatial information of each cell and pre-clustered gene expression. We then used a linear combination operation to merge the decorrelated latent embeddings, enhancing the discriminative power of the resulting embedding and clustering accuracy, thus facilitating

comprehensive analysis to provide profound insights into complex biological systems.

Our study demonstrates the effectiveness and robustness of SGAE in capturing tissue structures across different ST technology platforms. This superiority over other methods indicates the immense potential of SGAE as a reliable tool for analyzing ST datasets. Another notable strength of SGAE lies in its ability to accurately capture the heterogeneity present within ST datasets. The complexity and diversity of cell types within tissues pose significant challenges in accurately characterizing gene expression patterns. Notably, SGAE's embedding successfully captures the heterogenic information, enabling a more comprehensive understanding of the spatial organization of gene expression patterns within tissues. While SGAE has demonstrated its advantages in ST clustering, further validation across a wider range of ST datasets and biological systems is necessary to fully assess the generalizability of SGAE's performance.

In this study, we also applied SGAE to analyze the Drosophila 3D dataset and unravel the spatial domains during the E14-16, E16-18, and Larva L1 stages. We further compared the performance of SGAE with that of STAligner, a commonly used method developed for multi-slice ST analysis. By evaluating benchmark metrics, we consistently observed that SGAE outperformed STAligner in effectively grouping cells into biologically meaningful clusters. The superior clustering results of SGAE carry significant implications for the analysis of 3D tissue structure reconstruction. In conclusion, SGAE demonstrates its proficiency in spatial domain identification on spatial transcriptomics with moderate batch effect. For datasets with a high batch effect, it is recommended to integrate batch removal methods upstream of SGAE to effectively mitigate this issue. By accurately categorizing cells into reasonable groups, SGAE could contribute to a more precise characterization of the spatial organization of gene expression patterns. This is particularly important for understanding the complex processes underlying biological development and differentiation.

Methods

Notations and Problem Definition

An undirected graph is usually represented by $G = \{V, E\}$, where $V = \{v_1, v_2, \cdots, v_N\}$ and

E are the node and edge respectively. Each node v_i is characterized by a vector $\boldsymbol{x}_{\mathbf{i}} \in R^D$, where D is the dimension of the attribute. Then the graph can be characterized by the feature matrix $X \in R^{N \times D}$. The relation between each node is characterized by the adjacency matrix $A = (a_{ij})_{N \times N}$, where $a_{ij} = 1$ if v_i and v_j are connected by an edge, otherwise $a_{ij} = 0$. A degree matrix describes the number of edges connected to each node and can be expressed in a diagonal matrix $D = \text{diag}(d_1, d_2, \cdots, d_N) \in R^{N \times N}$, d_i is the degree of node v_i and calculated by $d_i = \sum_{(v_i, v_j) \in E} a_{ij}$. We normalize the adjacency matrix as $\widetilde{A} = D^{-1}(A + I)$ where $I \in R^{N \times N}$ is the identity matrix.

In this paper, we aim to train a Siamese graph encoder that embeds all nodes into the low-dimension latent space in an unsupervised manner. The resultant latent embedding can then be directly utilized to perform node clustering by clustering metrics such as K-means and Leiden.

The Overall Architecture of SGAE

The overall architecture of SGAE consists of Graph Distortion, Siamese Encoders, Siamese Decoders, and a reconstruction loss function.

Graph Distortion

We utilized two types of graph distortion including feature corruption and edge perturbation.

For feature corruption, which is the feature-level distortion, we apply Hadamard product to feature matrix and a random noise matrix generated from a Gaussian distribution, i.e., $\tilde{X} = X \odot N$, where $\odot$ means the Hadamard product and $N \sim N(1, 0.1)$.

For edge perturbation, which is the structure-level distortion, we adopt two types of distortion, i.e., edge-removing and graph diffusion. For the edge removal, we generated a mask matrix M according to the similarity matrix by calculating the pair-wise cosine similarity in the latent space, where the 10% of the lowest edges will be removed. The final adjacency matrix after edge removal is

$$A^m = D^{-\frac{1}{2}}\big((A \odot M) + I\big)D^{-\frac{1}{2}}$$

In the graph diffusion treatment, we used Personalized PageRank to calculate the normalized adjacency matrix into a graph diffusion matrix by following MVGRL method[28]:

$$A^d = \alpha\left(I - (1-\alpha)\left(D^{-\frac{1}{2}}(A+I)D^{-\frac{1}{2}}\right)\right)^{-1}$$

where $\alpha = 0.2$ as the teleport probability in a random walk.

Siamese Encoders

In order to reduce the utilization of space while learning richer cell representations, we constructed two same encoders based on Siamese network structure to encode cell features. The inputs of Siamese Encoders are graph $G_1 = (X_1, A_m)$ and graph $G_2 = (X_2, A_d)$. And the output is the embedding matrix H. First, we use two parameter-shared encoders to encode graph G_1 and graph G_2 respectively, and generate embedding matrices H_1 and H_2. The encoder in the l-th layer can be formulated as:

$$\mathrm{H}_1^{(l)} = \sigma\left(\widehat{\mathrm{A}_\mathrm{m}}\mathrm{H}_1^{(l-1)}\mathrm{W}_1^{(l)}\right) + \sigma\left(\mathrm{H}_1^{(l-1)}\mathrm{W}_2^{(l)} + \mathrm{b}^{(l)}\right)$$

$$\mathrm{H}_2^{(l)} = \sigma\left(\widehat{\mathrm{A}_\mathrm{d}}\mathrm{H}_2^{(l-1)}\mathrm{W}_1^{(l)}\right) + \sigma\left(\mathrm{H}_2^{(l-1)}\mathrm{W}_2^{(l)} + \mathrm{b}^{(l)}\right)$$

where, $\widehat{\mathrm{A}_\mathrm{m}} = \mathrm{D}_\mathrm{m}^{-\frac{1}{2}}(\mathrm{A}_\mathrm{m} + \mathrm{I})\mathrm{D}_\mathrm{m}^{-\frac{1}{2}}, \widehat{\mathrm{A}_\mathrm{d}} = \mathrm{D}_\mathrm{d}^{-\frac{1}{2}}(\mathrm{A}_\mathrm{d} + \mathrm{I})\mathrm{D}_\mathrm{d}^{-\frac{1}{2}}$, D_m and D_d are degree matrices of A_m and A_d, I is the identity matrix, $W_1^{(l)}$ and $W_2^{(l)}$ are weight matrices of encoders in the l-th layer, $b^{(l)}$ is the bias vector of encoder in the l-th layer, σ is the non-linear activate function, such as ReLU and Tanh. When layer $l = 1$, $H_1^{(l-1)} = X_1$. Ultimately, the decorrelated latent embeddings derived from two different views, namely H_1 and H_2, are merged using a linear combination operation. This amalgamation produces clustering-focused latent embeddings that can be effectively employed for clustering purposes, particularly through the utilization of the K-means algorithm.

Siamese Decoders

For SGAE, we construct a decoder based on graph convolutional neural networks, while reconstructing feature embeddings and adjacency matrices. The input is the embedding matrix

H, and the output is the original feature matrix X and the adjacency matrix A. Firstly, we use the graph convolutional neural network to decode the embedding H to generate a feature matrix $\widehat{H}$, and the calculation formula of the k layer decoder is as follows:

$$H^{(k)} = \sigma\left(D^{-\frac{1}{2}}(A + I)D^{-\frac{1}{2}}H^{(k-1)}W^{(k)}\right)$$

where D is the degree matrix of the matrix A, and $W^{(k)}$ is the parameter matrix of the k-th layer of the decoder. Then, taking inner product computation between the embedding matrix H and its transpose to generate the adjacency matrix $\widehat{A}$.

Reconstruction Loss Function

Finally, we calculate the feature matrix reconstruction loss L_{REC-F}, the calculation formula is as follows:

$$L_{REC-F} = \frac{1}{2N}\left|\left|AX - \widehat{H}\right|\right|_F^2$$

Calculate the adjacency matrix reconstruction loss L_{REC-A}, the calculation formula is as follows:

$$L_{REC-A} = \frac{1}{2N}\left|\left|A - \widehat{A}\right|\right|_F^2$$

The reconstruction loss L_{REC} is the sum of the feature matrix reconstruction loss and the adjacency matrix reconstruction loss, and the calculation formula is as follows:

$$L_{REC} = L_{REC-F} + L_{REC-A}$$

Redundant Reduction Module

In order to eliminate redundant information in node embedding and generate distinguishable embeddings for each node, the present invention designs a de-redundancy module, which eliminates redundant information from two levels: node level and feature level:

$$S_N = \frac{H_1 H_2^T}{||H_1||\ ||H_2||}$$

$$S_F = \frac{Z_1 Z_2^T}{||Z_1||\ ||Z_2||}$$

$$L_{RR} = L_{RR-N} + L_{RR-F}$$

Clustering Guidance Module

In order to effectively learn the feature embedding related to the clustering task, the present invention designs a clustering guidance module. Firstly, we pre-train the model, and use K-means to cluster the generated node embeddings. Secondly, we construct a clustering guidance loss L_C according to the node embedding matrix and the clustering result of the previous step: a) Compute the soft assignment matrix Q for all nodes and pre-trained cluster centers using the Student's t distribution. b) Generate the target distribution matrix P according to the soft allocation matrix Q , the element p_{ij} of the i row j column is calculated by the following formula:

$$p_{ij} = \frac{q_{ij}^2 / \sum_i q_{ij}}{\sum_{j'} (q_{ij'}^2 / \sum_i q_{ij'})}$$

Then compute the clustering guidance loss L_C using the KL divergence from the soft assignment, the target distribution and the pretrained soft assignment.

During training, the model is optimized by minimizing the loss function:

$$L = L_{REC} + L_C + L_{RR}$$

After the model training is completed, the main flow of data in the model inference process is as follows: firstly, the model is used to obtain the low-dimensional feature embedding H of cells, and then based on the learned embedding, K-means is used for clustering, and finally the cluster labels of all cells are obtained.

Clustering Refinement

SGAE also incorporates an optional clustering refinement step. During this step, SGAE analyzes the domain assignment of each spot and its neighboring spots. Specifically, for a given spot, the label that appears most frequently among its surrounding spots is assigned to that spot. The clustering refinement step was exclusively performed for the human DLPFC 10x Visium data.

Performance Evaluation

We use five indices to evaluate the quality of the clustering results: Adjusted Rand Index (ARI), Normalized Mutual Information (NMI), Fowlkes-Mallows Index (FMI), Adjusted Mutual_Infomation (AMI), and Moran's Index. These indices provide different perspectives on

the clustering performance. ARI measures the similarity of predicted types in the clusters, with a range from -1 to 1. NMI measures the relationship between variables and is normalized to a range of [0,1]. FMI calculates the geometric mean of pairwise precision and recall, also ranging from 0 to 1. AMI measures the similarity between the cluster assignments obtained from a clustering algorithm and the ground truth cluster assignments. The Moran's Index is used to assess spatial autocorrelation in the clustering results. Together, these indices offer a comprehensive evaluation of the clustering quality across various aspects.

Here are formulas and function APIs used to implement the indices.

ARI: sklearn.metrics.adjusted_rand_score

$$ARI = \frac{(TP + TN)}{C_N^2} \ (N = samples)$$

NMI: sklearn.metrics.normalized_mutual_info_score

$$MI(X,Y) = \sum_{i=1}^{|X|}\sum_{j=1}^{|Y|} P(i,j)\log\left(\frac{P(i,j)}{P(i)P(j)}\right)$$

$$H(X) = -\sum_{i=1}^{|X|} P(i)\log\big(P(i)\big); H(Y) = -\sum_{j=1}^{|Y|} P(j)\log\big(P(j)\big)$$

$$NMI(X,Y) = \frac{2MI(X,Y)}{H(X) + H(Y)}$$

FMI: sklearn.metrics.fowlkes_mallows_score

$$FMI = TP / sqrt((TP + FP) * (TP + FN))$$

AMI: sklearn.metrics.adjusted_mutual_info_score

$$AMI(X,Y) = \frac{MI(X,Y) - E\{MI(X,Y)\}}{1/2(H(X) + H(Y)) - E\{MI(X,Y)\}}$$

Moran's I: scanpy.metrics.morans_i

$$E[I] = -\frac{1}{n-1}; V[I] = E[I^2] - E[I^2] z_I = (I - E[I])/\sqrt{V[I]}$$

$$S_0 = \sum_{i=1}^{n}\sum_{j=1}^{n} \omega_{i,j}$$

$$I = \frac{n\sum_{i=1}^{n}\sum_{j=1}^{n}\omega_{i,j}\, z_i z_j}{S_0 \sum_{i=1}^{n} z_i^2}$$

Data Preprocessing

SGAE utilizes transcriptome-wide gene expression profiles with spatial coordinates as

input. The raw gene counts per spot are first normalized to the total counts per cell and then scaled through log-transformation. In the case of 3D Drosophila datasets, we did not employ any multi-slice integration method as there was little batch effect observed from the UMAP result. Principal component analysis (PCA) is then conducted on the gene expression data using the *sc.pp.pca()* function, and the top 50 principal components per spot are subsequently utilized as the default expression feature.

Identifying differentially expressed genes. Wilcoxon test implemented in SCANPY [29] is used to calculate differentially expressed genes for each spatial domain Benjamin-Hochberg adjustment correlation via *sc.tl.rank_genes_groups()*.

Spatial trajectory inference

We employed the PAGA algorithm [30] implemented in the SCANPY package to depict spatial trajectory. The PAGA trajectory and PAGA tree were inferred by the *scanpy.tl.paga()* function based on cell embedding generated by SGAE. Furthermore, *scanpy.tl.dpt()* was applied to estimate the pseudo time as well. To compare the performance of each clustering method with embedding, we calculate trajectory and pseudo time using methods above with same parameters settings.

Availability of supporting source code and requirements

Project name: SGAE

Project home page: https://github.com/STOmics/SGAE/

Operating system: Linux

Programming language: Python

License: MIT license

RRID: SCR_024803

Data Availability

Supporting data sets for this article are available via following databases: human dorsolateral prefrontal cortex 10x Visium dataset from spatialLIBD[31], mouse cortex

MERFISH dataset from Brain Image Library[32], mouse gastrulation seqFISH dataset from SpatialMouseAtlas[21], mouse olfactory bulb SLIDE-seq v2 dataset from Single Cell PORTAL[33], liver cancer Stereo-seq dataset and 3D Drosophila Stereo-seq dataset from CNGBdb[34], and adult mouse brain Stereo-seq dataset from Zenodo[35]. An archival version of SGAE can also be accessed in Software Heritage[36].

Abbreviations

ST: spatial transcriptomics, MERFISH: Multiplexed Error-Robust Fluorescence in situ Hybridization, seqFISH: sequential fluorescence in situ hybridization, scRNA-Seq: single-cell RNA sequencing, GCN: graph convolution network, GNN: graph neural network, DCRN: Dual Correlation Reduction Network, VGAE: Variational Graph Autoencoder, SNN: spatial neighbor network, UMAP: Uniform Manifold Approximation and Projection, KNN: K-nearest neighbors, ARI: Adjusted Rand Index, NMI: Normalized Mutual Information, FMI: Fowlkes-Mallows Index, DLPFC: dorsolateral prefrontal cortex, MI: Moran's Index.

Funding

This work is supported by National Natural Science Foundation for Young Scholars of China (32300526) and National Key R&D Program of China (2022YFC3400400)

Authors' Contributions

S.F. and Y.Z. conceived and designed the study. W.J., L.C., C.Y. and Y.R. proposed the SGAE model. L.C., L.H., C.Y., and Y.J. performed the data analysis. T.X. helped with the 3D reconstruction analysis. M.L. X.X, and Y.L. participated in the study discussions. L.C., L.H., C.Y., and S.F. wrote the manuscript.

Competing Interests

All authors declare no conflicts of interest in regard to this manuscript.

ACKNOWLEDGEMENTS

We thank Lidong Guo, Xiaobin Liu for their help to the manuscript. This work is part of the

"SpatioTemporal Omics Consortium" (STOC) paper package. A list of STOC members is available at: http://sto-consortium.org. We acknowledge the Stomics Cloud platform to provide convenient ways for analyzing spatial omics datasets. We acknowledge the CNGB Nucleotide Sequence Archive (CNSA) of China National GeneBank DataBase (CNGBdb) for maintaining the MOSTA and Flysta3D database.

References

1. Park HE, Jo SH, Lee RH, Macks CP, Ku T, Park J, et al. Spatial Transcriptomics: Technical Aspects of Recent Developments and Their Applications in Neuroscience and Cancer Research. Adv Sci (Weinh). 2023;10 16:e2206939. doi:10.1002/advs.202206939.
2. Larsson L, Frisen J and Lundeberg J. Spatially resolved transcriptomics adds a new dimension to genomics. Nat Methods. 2021;18 1:15-8. doi:10.1038/s41592-020-01038-7.
3. Chen KH, Boettiger AN, Moffitt JR, Wang S and Zhuang X. RNA imaging. Spatially resolved, highly multiplexed RNA profiling in single cells. Science. 2015;348 6233:aaa6090. doi:10.1126/science.aaa6090.
4. Shah S, Takei Y, Zhou W, Lubeck E, Yun J, Eng CL, et al. Dynamics and Spatial Genomics of the Nascent Transcriptome by Intron seqFISH. Cell. 2018;174 2:363-76 e16. doi:10.1016/j.cell.2018.05.035.
5. Rodriques SG, Stickels RR, Goeva A, Martin CA, Murray E, Vanderburg CR, et al. Slide-seq: A scalable technology for measuring genome-wide expression at high spatial resolution. Science. 2019;363 6434:1463-7. doi:10.1126/science.aaw1219.
6. Stahl PL, Salmen F, Vickovic S, Lundmark A, Navarro JF, Magnusson J, et al. Visualization and analysis of gene expression in tissue sections by spatial transcriptomics. Science. 2016;353 6294:78-82. doi:10.1126/science.aaf2403.
7. Chen A, Liao S, Cheng M, Ma K, Wu L, Lai Y, et al. Spatiotemporal transcriptomic atlas of mouse organogenesis using DNA nanoball-patterned arrays. Cell. 2022;185 10:1777-92 e21. doi:10.1016/j.cell.2022.04.003.
8. Bressan D, Battistoni G and Hannon GJ. The dawn of spatial omics. Science. 2023;381 6657:eabq4964. doi:10.1126/science.abq4964.
9. Burgess DJ. Spatial transcriptomics coming of age. Nat Rev Genet. 2019;20 6:317. doi:10.1038/s41576-019-0129-z.
10. Long Y, Ang KS, Li M, Chong KLK, Sethi R, Zhong C, et al. Spatially informed clustering, integration, and deconvolution of spatial transcriptomics with GraphST. Nat Commun. 2023;14 1:1155. doi:10.1038/s41467-023-36796-3.
11. Li J, Chen S, Pan X, Yuan Y and Shen H-B. Cell clustering for spatial transcriptomics data with graph neural networks. Nature Computational Science. 2022;2 6:399-408. doi:10.1038/s43588-022-00266-5.
12. Hu J, Li X, Coleman K, Schroeder A, Ma N, Irwin DJ, et al. SpaGCN: Integrating gene expression, spatial location and histology to identify spatial domains and spatially variable genes by graph convolutional network. Nat Methods. 2021;18 11:1342-51. doi:10.1038/s41592-021-01255-8.

13. Dong K and Zhang S. Deciphering spatial domains from spatially resolved transcriptomics with an adaptive graph attention auto-encoder. Nat Commun. 2022;13 1:1739. doi:10.1038/s41467-022-29439-6.

14. Liu Y, Tu W, Zhou S, Liu X, Song L, Yang X, et al. Deep Graph Clustering via Dual Correlation Reduction. arXiv e-prints. 2021:arXiv:2112.14772. doi:10.48550/arXiv.2112.14772.

15. Kipf TN and Welling M. Variational Graph Auto-Encoders. arXiv e-prints. 2016:arXiv:1611.07308. doi:10.48550/arXiv.1611.07308.

16. Page L, Brin S, Motwani R and Winograd T. The PageRank Citation Ranking : Bringing Order to the Web. In: *The Web Conference* 1999.pp. 567-574.

17. Zbontar J, Jing L, Misra I, LeCun Y and Deny SpJae-p. Barlow Twins: Self-Supervised Learning via Redundancy Reduction. *arXiv e-prints.* 2021. doi:10.48550/arXiv.2103.03230.

18. Lubeck E, Coskun AF, Zhiyentayev T, Ahmad M and Cai L. Single-cell in situ RNA profiling by sequential hybridization. Nature Methods. 2014;11 4:360-1. doi:10.1038/nmeth.2892.

19. Stickels RR, Murray E, Kumar P, Li J, Marshall JL, Di Bella DJ, et al. Highly sensitive spatial transcriptomics at near-cellular resolution with Slide-seqV2. Nat Biotechnol. 2021;39 3:313-9. doi:10.1038/s41587-020-0739-1.

20. Maynard KR, Collado-Torres L, Weber LM, Uytingco C, Barry BK, Williams SR, et al. Transcriptome-scale spatial gene expression in the human dorsolateral prefrontal cortex. Nat Neurosci. 2021;24 3:425-36. doi:10.1038/s41593-020-00787-0.

21. Lohoff T, Ghazanfar S, Missarova A, Koulena N, Pierson N, Griffiths JA, et al. Integration of spatial and single-cell transcriptomic data elucidates mouse organogenesis. Nat Biotechnol. 2022;40 1:74-85. doi:10.1038/s41587-021-01006-2.

22. Zhang M, Eichhorn SW, Zingg B, Yao Z, Cotter K, Zeng H, et al. Spatially resolved cell atlas of the mouse primary motor cortex by MERFISH. Nature. 2021;598 7879:137-43. doi:10.1038/s41586-021-03705-x.

23. Wu L, Yan J, Bai Y, Chen F, Zou X, Xu J, et al. An invasive zone in human liver cancer identified by Stereo-seq promotes hepatocyte–tumor cell crosstalk, local immunosuppression and tumor progression. Cell Research. 2023;33 8:585-603. doi:10.1038/s41422-023-00831-1.

24. Waltman L and Van Eck NJJTEpjB. A smart local moving algorithm for large-scale modularity-based community detection. The European physical journal B. 2013;86:1-14.

25. Shen R, Liu L, Wu Z, Zhang Y, Yuan Z, Guo J, et al. Spatial-ID: a cell typing method for spatially resolved transcriptomics via transfer learning and spatial embedding. Nat Commun. 2022;13 1:7640. doi:10.1038/s41467-022-35288-0.

26. Zhou X, Dong K and Zhang S. Integrating spatial transcriptomics data across different conditions, technologies, and developmental stages. *Nature Computational Science.* 2022:2022.12.26.521888. doi:10.1101/2022.12.26.521888 %J bioRxiv.

27. Wang M, Hu Q, Lv T, Wang Y, Lan Q, Xiang R, et al. High-resolution 3D spatiotemporal transcriptomic maps of developing Drosophila embryos and larvae. Dev Cell. 2022;57 10:1271-83 e4. doi:10.1016/j.devcel.2022.04.006.

28. Hassani K and Hosein Khasahmadi A. Contrastive Multi-View Representation Learning on Graphs. arXiv e-prints. 2020:arXiv:2006.05582. doi:10.48550/arXiv.2006.05582.

29. Wolf FA, Angerer P and Theis FJ. SCANPY: large-scale single-cell gene expression data analysis. Genome Biol. 2018;19 1:15. doi:10.1186/s13059-017-1382-0.

30. Wolf FA, Hamey FK, Plass M, Solana J, Dahlin JS, Göttgens B, et al. PAGA: graph abstraction reconciles clustering with trajectory inference through a topology preserving map of single cells. Genome Biology. 2019;20 1:59. doi:10.1186/s13059-019-1663-x.
31. Pardo B, Spangler A, Weber LM, Page SC, Hicks SC, Jaffe AE, et al. spatialLIBD: an R/Bioconductor package to visualize spatially-resolved transcriptomics data. BMC Genomics. 2022 Jun 10;23(1):434. doi: 10.1186/s12864-022-08601-w.
32. Benninger K, Hood G, Simmel D, Tuite L, Wetzel AW, Ropelewski AJ, et al. Cyberinfrastructure of a Multi-Petabyte Microscopy Resource for Neuroscience Research. In: *PEARC '20: Practice and Experience in Advanced Research Computing* 2020. https://doi.org/10.1145/3311790.3396653
33. Single Cell Portal: an interactive home for single-cell genomics data. bioRxiv. 2023 Jul 17:2023.07.13.548886. doi: 10.1101/2023.07.13.548886..
34. Chen FZ, You LJ, Yang F, Wang LN and Wei XFJH. CNGBdb: China National GeneBank DataBase. 2020;42 8:799-809.
35. Shen R, Liu L, Wu Z, Zhang Y, Yuan Z, Guo J, et al. Data from: Application of Spatial-ID to large field mouse brain hemisphere dataset measured by Stereo-seq. Zenodo, 2022. https://doi.org/10.5281/zenodo.7340795
36. Fang S, Cao L, Yang C, Hu L et al. (2023) SGAE: Deciphering spatial domains from spatially resolved transcriptomics with Siamese Graph Autoencoder (Version 1). [Computer software]. Software Heritage, https://archive.softwareheritage.org/browse/snapshot/19c3ac3c492b5b4c6aca5451eeea9efb52a3ad9d/directory/?origin_url=https://github.com/STOmics/SGAE

Figure legends

Figure 1. An overview of SGAE framework. SGAE algorithm consists of three key modules. Firstly, the graph distortion module generates two distorted graphs by introducing both attribute and graph disturbances. Secondly, the encoder module generates two sets of representations for each sample. Thirdly, the redundant reduction module ensures that the same sample within the two distorted graphs has identical representations at both the feature and sample levels. Lastly, the discriminative representations are applied to clustering algorithms such as k-means to decipher spatial domains.

Figure 2. SGAE exhibited high effectiveness and robustness in spatial domain exploration. (A-D) Visualization of clustering results from SGAE, SpaGCN, GraphST, STAGATE, Leiden and annotation. (A) Human dorsolateral prefrontal cortex (DLPFC) 10x Visium dataset, (B) Mouse gastrulation seqFISH dataset, (C) Mouse cortex MERFISH dataset, (D) Mouse olfactory bulb SLIDE-seq v2 dataset. (E-G) Benchmark metrics comparison of SGAE against SpaGCN,

GraphST, STAGATE and Leiden. (E) Boxplot of ARI, FMI and NMI for 12 DLPFC 10x Visium datasets.(F) Mouse gastrulation seqFISH dataset, (G) Mouse cortex MERFISH dataset.

Figure 3. SGAE unraveled spatial domains and provided discriminative representations. (A) Visualization of human adult brain clustering results from SGAE, SpaGCN, STAGATE, CCST, and GraphST. (B) Subclustering results of DGGRC2, TEGLU24 and HBGLU from SGAE, SpaGCN, STAGATE, CCST, and GraphST. (C) Benchmark metrics comparison of SGAE against SpaGCN, STAGATE, CCST, and GraphST. (D) Boxplot of Moran's Index comparison of SGAE against SpaGCN, STAGATE, CCST, and GraphST. (E) UMAP visualization of embedding from SGAE, SpaGCN, STAGATE and GraphST. (F) Boxplot of ANOVA F score of pseudo-time calculated from embedding provided by PCA, CCST, STAGATE and GraphST.

Figure 4. SGAE enhanced complex spatial domain dissection in 3D Drosophila Embryo. (A) 2D visualization of Drosophila Embryo clustering results at different stages (E14-16, E16-18, and L1) from SGAE and STAligner. (B) Benchmark metrics comparison of SGAE, Leiden and STAligner. (C) 3D visualization of Drosophila Embryo. The first row showed the marker genes of Drosophila Embryo at different stages, while the last three rows displayed the meshes generated by SGAE, STAligner and Leiden respectively.

Supplementary Figure 1. SGAE reached good performance on complex and heterogenous liver cancer sample. (A) H&E staining of liver cancer sample. Manually added line indicate the border of tumor and healthy tissue. (B) Clustering result of SGAE and other methods. (C) Moran's Index of the clustering results of SGAE and other methods. (D) Spatial map of the expression of VIM (E) The most likely clusters associated with fibroblasts identified using SGAE and other methods, determined by the expression of VIM.

Supplementary Figure 2. Less batch effect detected in 3D Drosophila embryos. UMAP visualization of 3D Drosophila embryos. Left : color in cell type annotation, Right : color in slices of sample. (A) E14-16. (B) E16-18. (C) L1.

Figure 1 Click here to access/download;Figure;Figure1.tif

Figure 1

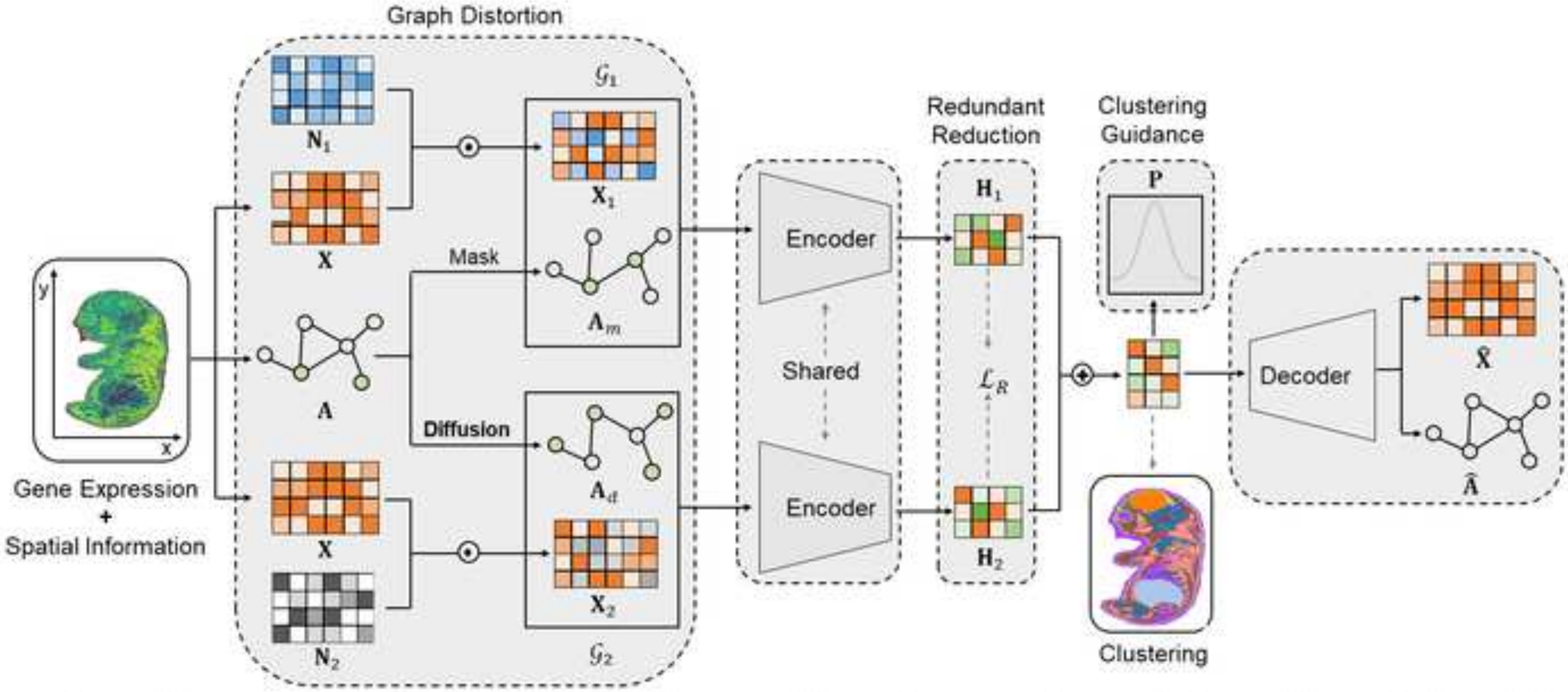

$\mathbf{A}$: Adjacency matrix, $\mathbf{X}$: Feature Matrix, $\mathcal{G}_1$, $\mathcal{G}_2$: Graph, $\mathbf{N}_1$, $\mathbf{N}_2$: Gauss Noise Matrix, $\mathbf{H}$: Embedding Matrix, $\mathbf{P}$: Target distribution, $\odot$: Hadamard Product, $\oplus$: Addition, $\mathcal{L}$: Loss

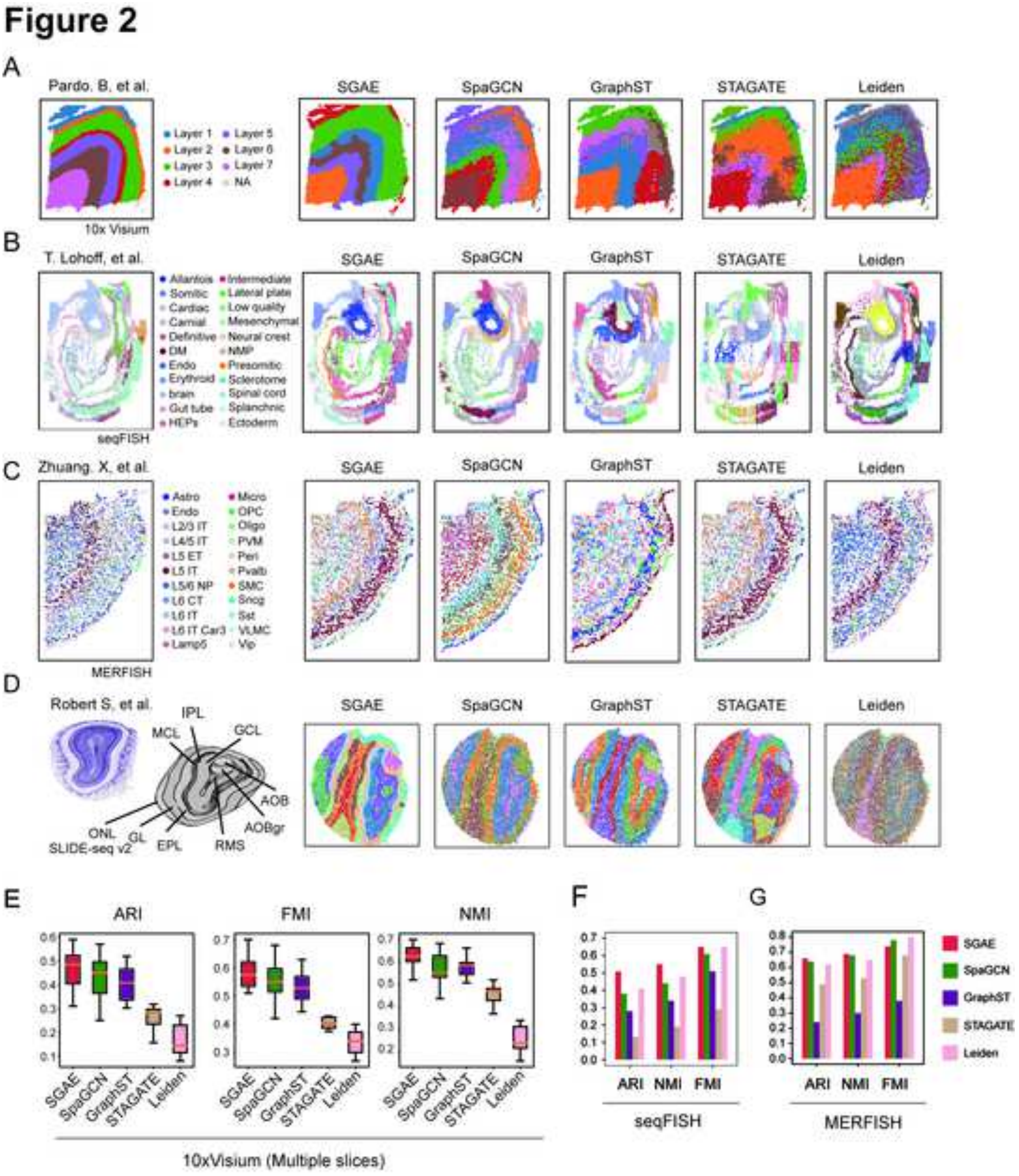
Figure 2
A
Pardo. B. et al.
10x Visium
Layer 1
Layer 2
Layer 3
Layer 4
Layer 5
Layer 6
Layer 7
NA
SGAE
SpaGCN
GraphST
STAGATE
Leiden
B
T. Lohoff, et al.
seqFISH
Allantois
Somitic
Cardiac
Cranial
Definitive
DM
Endo
Erythroid
brain
Gut tube
HEPs
Intermediate
Lateral plate
Low quality
Mesenchymal
Neural crest
NMP
Presomitic
Sclerotome
Spinal cord
Splanchnic
Ectoderm
C
Zhuang. X, et al.
MERFISH
Astro
Endo
L2/3 IT
L4/5 IT
L5 ET
L5 IT
L5/6 NP
L6 CT
L6 IT
L6 IT Car3
Lamp5
Micro
OPC
Oligo
PVM
Peri
Pvalb
SMC
Sncg
Sst
VLMC
Vip
D
Robert S, et al.
SLIDE-seq v2
IPL
MCL
GCL
AOB
AOBgr
ONL
GL
EPL
RMS
E
ARI
FMI
NMI
10xVisium (Multiple slices)
F
seqFISH
G
MERFISH
ARI NMI FMI

Figure 4

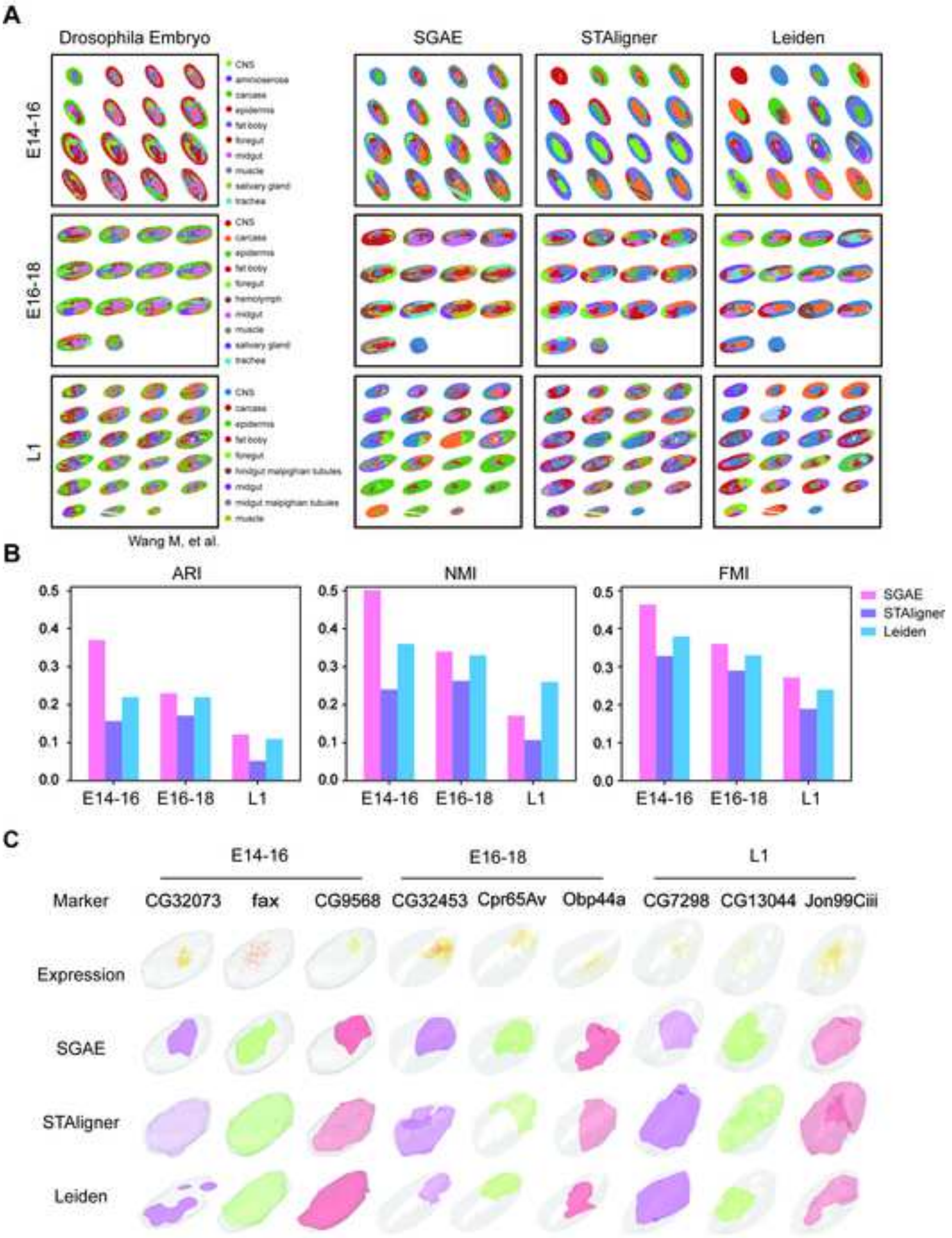
A
Drosophila Embryo
SGAE
STAligner
Leiden
E14-16
E16-18
L1
CNS
amnioserosa
carcass
epidermis
fat body
foregut
midgut
muscle
salivary gland
trachea
hemolymph
hindgut malpighian tubules
midgut malpighian tubules
Wang M. et al.
B
ARI
NMI
FMI
SGAE
STAligner
Leiden
0.0
0.1
0.2
0.3
0.4
0.5
E14-16
E16-18
L1
C
E14-16
E16-18
L1
Marker
CG32073
fax
CG9568
CG32453
Cpr65Av
Obp44a
CG7298
CG13044
Jon99Ciii
Expression
SGAE
STAligner
Leiden

Figure 3

Click here to access/download;Figure;Figure3.tif

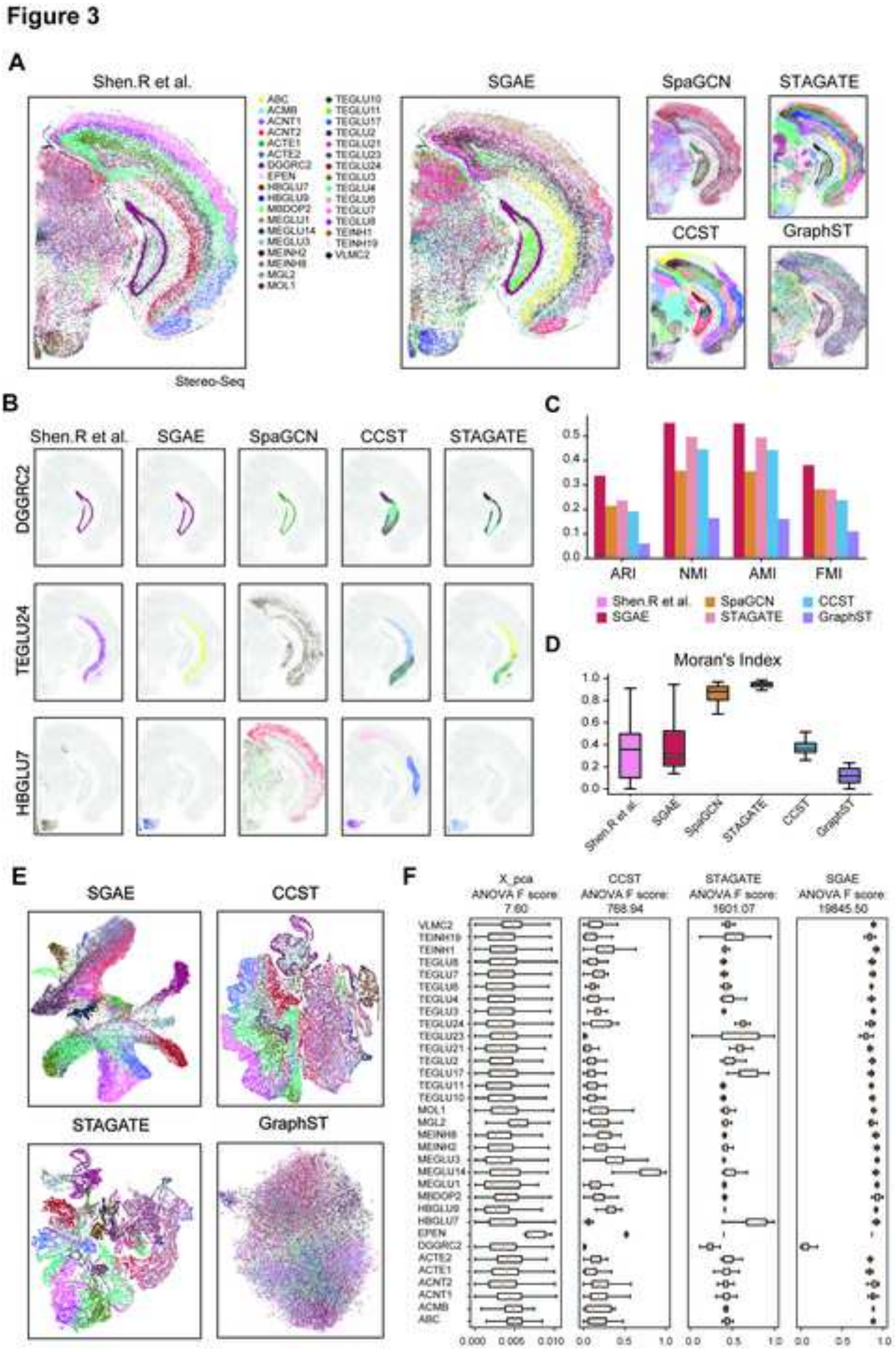

Supplementary Figure 1 Click here to access/download;Figure;SuppFig1.tif

Supplementary Fig. 1

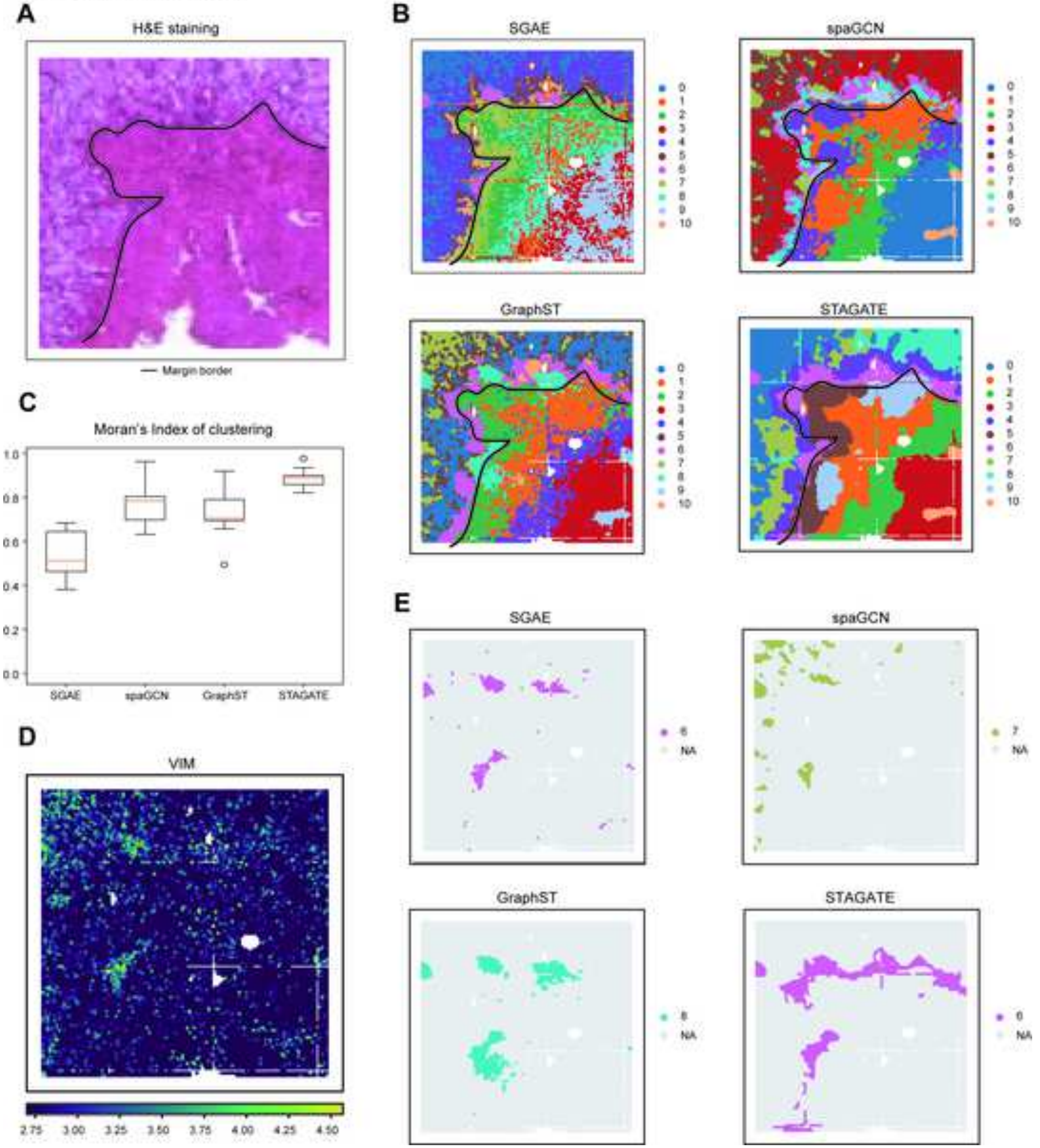

Supplementary Figure 2

Click here to access/download;Figure;SuppFig2.tif

Supplementary Fig. 2

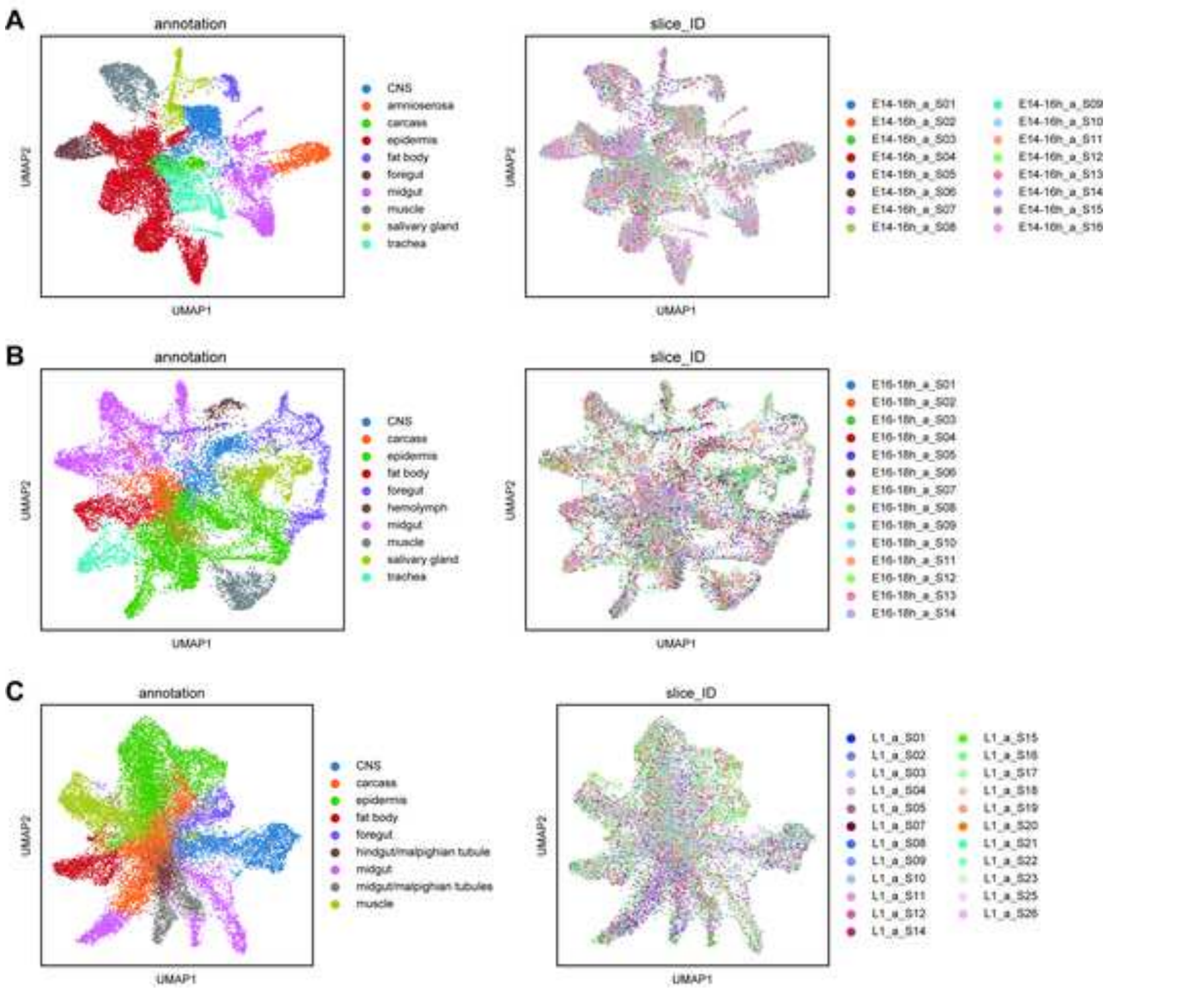

 Click here to access/download;Personal Cover;SGAE_Response_Letter.docx

Dear Editor,

Thank you for handling our manuscript entitled "Deciphering spatial domains from spatially resolved transcriptomics with Siamese Graph Autoencoder" and providing us with an opportunity to revise our work. We are delighted to receive positive remarks from both the editors and reviewers regarding our study. We would also like to express our sincere appreciation for the constructive comments provided by both the editors and reviewers, which have greatly strengthened our work.

We are grateful to the editors for sharing the comments of all three reviewers with us. In this revision, we have diligently addressed all the suggested experiments proposed by reviewers. Please find our point-by-point responses to the editors' comments listed below.

Sincerely,

Shuangsang Fang
fangshuangsang@genomics.cn

Reviewer’s comments:

Reviewer #1: This paper introduces SGAE, a novel method designed for the detection of spatial domains in spatial transcriptomics (ST). The authors utilize various public datasets and demonstrate that SGAE enhances representation discrimination, outperforming other spatial transcriptomics clustering software in clustering index evaluations. The study hol ds significant interest, as the application of SGAE has the potential to offer profound insi ghts into complex biological systems.

Response: We greatly appreciate the reviewer's positive remarks on our study and highly

value the reviewer's comments about our study.

While the authors effectively showcase the efficacy and robustness of SGAE across vario us real datasets, it would be intriguing to assess SGAE's performance and compare it with other ST methods under different sequencing depths and clustering parameters. Addition ally, a comparative analysis of maximum memory usage and runtime among various ST methods could provide valuable insights.

Response: We greatly appreciate the suggestions from the reviewers. To validate the robustness and accuracy of the SGAE algorithm, we conducted comprehensive tests on diverse datasets obtained from various ST methods. Importantly, we ensured that no special parameter adjustments were made during clustering process. The parameter table for testing on different datasets is also included in the Github repository (https://github.com/STOmics/SGAE).

Additionally, we have also performed statistical analysis on the memory usage and runtime of SGAE across different datasets (**see Table below**). In the architecture of SGAE, it involves pretrain models to ensure the clustering results, which may lead to the lower performance in terms of memory usage and runtime. However, based on the pretrain models designed in SGAE, the clustering results are more robust when SGAE applied on different datasets generated by different platforms (see Figure 2).

Table GPU memory, memory and time consumptions of SGAE and alternative methods

	GPU memory (Mb)					memory (Mb)					time (s)				
	SGAE	GraphST	spaGCN	STA-GATE	Leiden	SGAE	GraphST	spaGCN	STA-GATE	Leiden	SGAE	GraphST	spaGCN	STA-GATE	Leiden
DLPFC_151507	3033	1903	/	3009	/	1656	3701	1048	2998	2527	2980	56	71	26	140
DLPFC_151508	3703	2225	/	3013	/	1776	4951	825	3011	3217	3012	53	64	26	145
DLPFC_151509	3033	2991	/	3273	/	2278	5908	1205	3008	3985	2906	64	83	29	171
DLPFC_151510	3033	2991	/	3169	/	2287	6266	1153	3007	4710	2694	45	111	27	166
DLPFC_151669	3708	3003	/	2829	/	2287	7263	772	3005	5181	2754	42	76	23	150
DLPFC_151670	3033	3011	/	2725	/	2287	7761	878	2996	5722	2386	43	41	22	182
DLPFC_151671	3033	3011	/	3045	/	2287	8271	1075	3016	6435	2214	31	81	26	189
DLPFC_151672	3033	3085	/	2977	/	2287	9351	1048	3007	7072	2190	36	126	25	159
DLPFC_151673	3033	3085	/	2877	/	1328	9851	983	3026	7602	2688	40	71	24	128
DLPFC_151674	3033	3085	/	2977	/	1336	10386	734	3024	8202	2226	40	81	25	156
DLPFC_151675	3033	3085	/	2809	/	1366	10785	1114	3026	8798	2406	40	39	22	152
DLPFC_151676	3033	3275	/	2777	/	1336	11852	707	3029	9352	2590	40	59	23	140
Merfish	6272	1544	/	1710	/	1154	1228	425	2908	8328	3530	46	42	11	14
seqfish	12272	4772	/	3452	/	2164	7065	8519	13434	8349	14690	153	486	21	31
slide-seq	3275	6514	/	8876	/	3286	11192	12025	2979	13229	12486	701	538	101	160

In the "Results" section, enhancing the data description would contribute to a more comp elling presentation.

Response: We are grateful for the reviewer's professional comments. We have added detailed data description in the "Results" section (Line 120-129, 192-194).

Please provide the explanations of error bars in the figure legends.

Response： We much appreciate the reviewer's careful reading. We have added detailed explanations of statistics in the figure legend.

Furthermore, it is recommended to standardize specialized terms, such as 'F-score,' for co nsistency throughout the paper.

Response： We thank the reviewer for his/her professional suggestion. We have checked and revised the manuscript thoroughly to ensure specialized terms consistency.

Reviewer #2: The paper entitled "Deciphering spatial domains from spatially resolved tra nscriptomics with Siamese Graph Autoencoder" by Lei Cao, et al. explores an innovative framework called SGAE for spatial domain identification in spatial transcriptomics (ST) data analysis. This framework addresses the limitations of existing methods by incorporat ing the power of Siamese Graph Autoencoder (SGAE) to improve representation discrimi nation. The article also highlights the potential of SGAE in enhancing the accuracy of ide ntifying 3D Drosophila embryonic structures. Nevertheless, several significant concerns r emain regarding the author's stated conclusion.

Response: We are deeply grateful for the reviewer's positive feedback regarding our study and genuinely appreciate the valuable comments and insights.

1.The author mentioned "GNN-based methods suffer from representation collapse, where in all spatial spots are projected onto a singular representation." and "SGAE mitigates the information correlation at the both sample and feature level, thus improving the represent

ation discrimination." However, the author did not provide enough evidence to demonstrate the ability of SGAE in solving representation collapse, nor did they prove that the GNN approach indeed leads to such problems. More analysis evidence required to support the role of SGAE in representation collapse. Additionally, it would be helpful to understand the impact of representation collapse on downstream bioinformatic analysis.

Response: We sincerely appreciate the reviewer's insightful concerns. In response to the comments, we conducted additional analyses and obtained further evidence to support the role of SGAE in mitigating representation collapse (Figure below). Heatmap of pairwise Pearson’s correlation between center embedding of cell types illustrates a more discriminative embedding generated by SGAE compared to GAE (Graph Autoencoder) and AE (Autoencoder). Based on these findings, we provide empirical evidence that supports the ability of SGAE to mitigate representation collapse and improve representation discrimination.

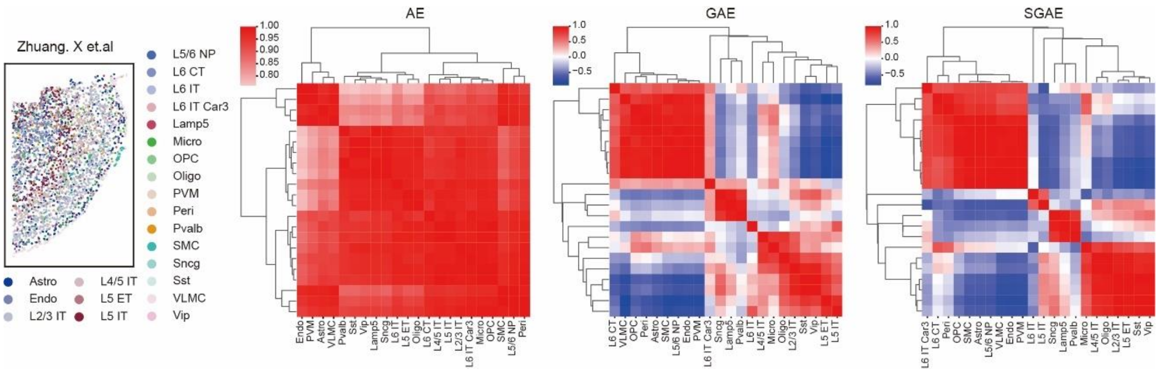

2.Did the Siamese architecture make a difference on the performance of spatial domain identification? Further ablation experiments are needed to demonstrate the effectiveness of each module in the SGAE architecture.

Response: We appreciate the reviewer's comment and agree that further ablation experiments would be valuable in assessing the effectiveness of SGAE architecture. We further compared the performance of the AE (Autoencoder) architecture, GAE (Graph

Autoencoder) architecture with the SGAE (Siamese Graph Autoencoder) architecture to evaluate the impact of the Siamese architecture on spatial domain identification. The results demonstrate that the SGAE architecture outperforms the GAE architecture in terms of spatial domain identification (Figure below). This suggests that the incorporation of the Siamese architecture in SGAE has a positive impact on the performance of spatial domain identification.

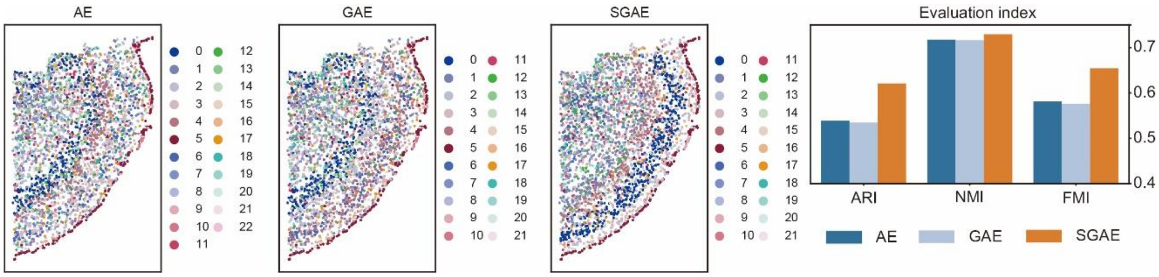

3.The author only analyzed the trajectory of one mouse brain sample. This is still far from proving that the embeddings generated by SGAE can exhibit better performance in down stream applications. Does the result of trajectory inference correspond to the actual biolo gical development process?

Response: We much appreciate the reviewer's comments and acknowledge the limitations of our study in terms of analyzing the pseudo-time of mouse brain sample among cell types. To further explain the rationality the pseudo-time result based on embedding of SGAE, we analysis the pseudo-time of cell types along a developmental trajectory from TEGLU24 to TEGLU7. As a result, SGAE successfully revealed the sustained upward trend of pseudo-time, providing evidence of the performance and utility of SGAE in capturing the underlying developmental processes.

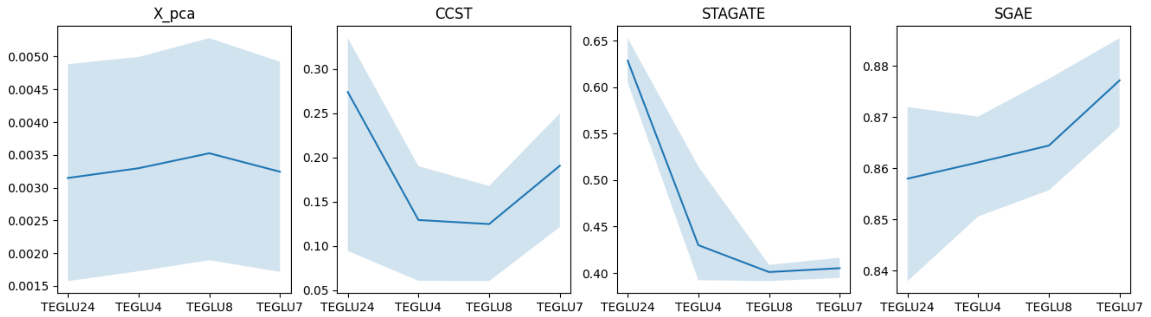

4.Did author adapt proper parameter on the other candidate models? Or is there any different on the preprocess of SGAE and other candidate method? Some of the candidate methods showed significantly poorer results compared to what was reported in corresponding paper, For example the Fig2 A.

Response: We much appreciate the reviewer for his/her careful reading. We did not perform any specific parameter tuning for the other candidate models and performed analysis using the default parameters provided by each model. It is reasonable for these methods to use the most proper results to show the performance and illustrate the study in their corresponding papers.

5.The author mainly used K-means to generate a pseudo-label when pre-clustering. While Louvain or Leiden is used to preform clustering on cell embeddings. Is there any preference of SGAE on choose the pre-cluster method and final cluster method?

Response: We much appreciate the reviewer for his/her insightful comments.

Actually, all clustering methods like Louvain, Leiden and K-means can be chosen as the clustering method. To make the parameters of application of SGAE the same, we chose K-means as the default clustering method.

6.It would be helpful to include a comprehensive hyperparameters table in order to elucidate the relative impact of each parameter on the model's performance. This table should also provide insights into the potential consequences of adjusting these parameters to higher or lower values.

Response: We sincerely thank the reviewer for his/her helpful suggestions. We have incorporated a comprehensive hyperparameters table in our Github repository (https://github.com/STOmics/SGAE).

7.The author declaimed little batch effect detected on 3D Drosophila datasets. Is there any evidence? How would SGAE performance on multi-slice datasets with high batch effect.

Response: We sincerely appreciate the reviewer's professional comments. The incorporation of UMAP analysis supports that different slices within each stage exhibit strong mixing, indicating minimal batch effects in the 3D Drosophila datasets (see Supplementary Figure 2).

Supplementary Fig. 2

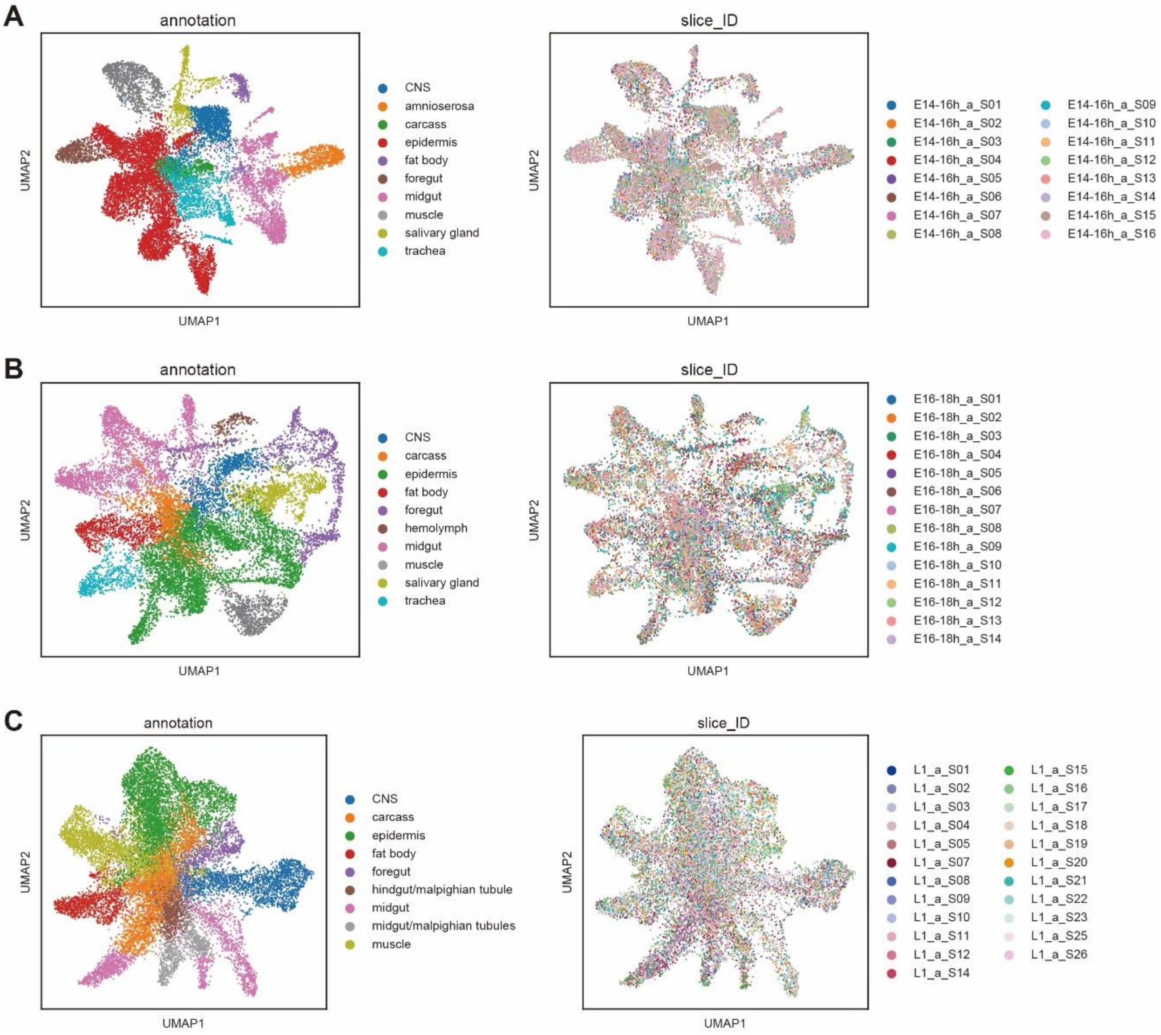

When constructing the SGAE framework, our primary focus was to achieve better ST clustering performance, without specifically considering solutions for addressing batch effects among multiple ST slices. As suggested in the discussion, if the data exhibits substantial batch effects, it would be advisable to first apply batch effect removal techniques to the datasets before utilizing SGAE for clustering analysis. This sequential approach can help mitigate the impact of batch effects and enhance the accuracy of subsequent analyses performed with SGAE.

Reviewer #3: In the manuscript entitled 'Deciphering spatial domains from spatially resolved transcriptomics with Siamese Graph Autoencoder', Cao et al developed a new computational framework for spatial domain identification in spatial transcriptomics data. The new framework (SGAE) incorporates the power of Siamese Graph Autoencoder, which mitigates the information correlation at both sample and feature levels. Through a series of benchmarking based on ST datasets generated from different platforms, the authors show that SGAE outperforms other ST clustering methods. Particularly, SGAE has shown its potential for extension and application in multi-slice 3D reconstruction and tissue structure investigation.

Overall, the manuscript describes a useful computational framework for ST data clustering and spatial domain identification. The method is sound and the manuscript is well-written.

Response: We are incredibly appreciative of the reviewer's positive feedback on our study and we highly value their comments.

A few points need to be addressed before publication.

1.The authors used datasets from different ST platforms and various tissues to examine the performance of SGAE and benchmark against other ST clustering methods. The ST data used in the current manuscript are all from tissues with clear structures such as the mouse cortex and human dorsolateral prefrontal cortex. However, to show the versatility of SGAE, can the authors test its performance when handling more complex and heterogenous tissues like tumors. It would be useful to show the results.

Response: We really thank the reviewer for his/her professional suggestions. We also assessed SGAE clustering performance over liver cancer samples (CNSA, accession code:

CNP0002199) (see Supplementary Figure 1). The application of SGAE resulted in a clearer and more accurate identification of the margin border based on H&E staining (Supplementary Fig 1A-B). Notably, SGAE also detected clusters consisting of discrete spots located in different positions, reflecting the heterogeneous nature of the tumor tissue. To assess the spatial correlation of the clustering results, we computed the Moran's Index. The Moran's Index revealed that alternative methods tended to overutilize spatial information and identify clusters in blocks (Supplementary Fig 1C). To further evaluate the accuracy of the clustering results obtained by these tools, we focused on the rare cell type fibroblast and used VIM as a marker gene for fibroblasts. We visualized the spatial distribution of VIM and compared it with the most probable cluster identified by each of the methods. The results showed that Cluster 6 in SGAE exhibited a higher similarity to the spatial expression of VIM compared to other methods (Supplementary Fig 1D-E).

Based on the above results, we confirmed the potential of SGAE for handling more complex and heterogenous tissues.

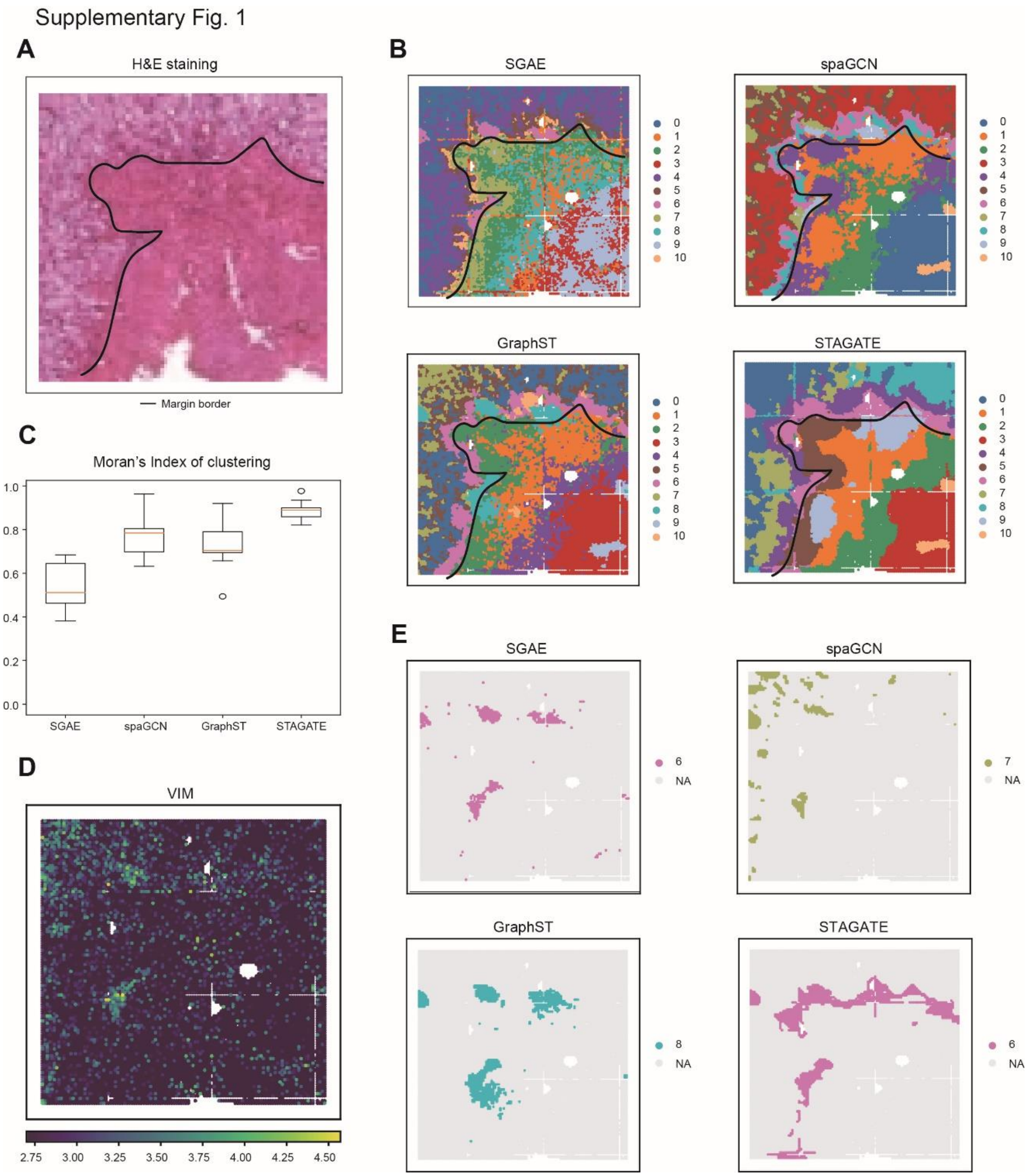

2. The authors benchmarked SGAE based on several metrics including ARI, NMI, and FMI. However, the current description of these benchmarking metrics and how exactly benchmarking has been conducted (in the method section) is rudimentary in the manuscript. More detailed information in both the **main text** and the **method section** is essential for a better understanding of the method and manuscript.

Response: We are really grateful for the reviewer’s professional suggestions. We have added more benchmarking details in the revised manuscript (Line 407-423).

3. More tutorials regarding how to use SGAE need to be included on the Github page.

Response: We much appreciate the reviewer’s helpful suggestion. We have incorporated a tutorial on our Github page. This tutorial aims to provide additional guidance and support to users interested in our algorithm.

4.The current resolution of all figures in the manuscript is poor, which largely influences t he reading. Can authors improve the figure resolution in revision?

Response: We are thankful for the reviewer’s careful reading. We have also provided figures with “vector graphic” format in our submission.
