# Supplementary material for: Deciphering spatial domains from spatially resolved transcriptomics with Siamese graph autoencoder
Source: Gigascience. 2024 Feb 20;13:giae003. doi: 10.1093/gigascience/giae003 (PMC10939418; doi:10.1093/gigascience/giae003)
Supplement: giae003_Response_to_Reviewer_Comments_Original_Submission [file giae003_response_to_reviewer_comments_original_submission.pdf]

Author's Response To Reviewer Comments

Dear Editor,

Thank you for handling our manuscript entitled "Deciphering spatial domains from spatially resolved transcriptomics with Siamese Graph Autoencoder" and providing us with an opportunity to revise our work. We are delighted to receive positive remarks from both the editors and reviewers regarding our study. We would also like to express our sincere appreciation for the constructive comments provided by both the editors and reviewers, which have greatly strengthened our work.

We are grateful to the editors for sharing the comments of all three reviewers with us. In this revision, we have diligently addressed all the suggested experiments proposed by reviewers. Please find our point-by-point responses to the editors' comments in the cover letter.

Sincerely,
Shuangsang Fang
fangshuangsang@genomics.cn
