# Supplementary material for: Deciphering spatial domains from spatially resolved transcriptomics with Siamese graph autoencoder
Source: Gigascience. 2024 Feb 20;13:giae003. doi: 10.1093/gigascience/giae003 (PMC10939418; doi:10.1093/gigascience/giae003)
Supplement: giae003_Reviewer_2_Report_Original_Submission — Jia Song -- 12/12/2023 [file giae003_reviewer_2_report_original_submission.pdf]

Reviewer Report

Title: Deciphering spatial domains from spatially resolved transcriptomics with Siamese Graph Autoencoder

Version: Original Submission **Date:** 12/12/2023

Reviewer name: Jia Song

Reviewer Comments to Author:

The paper entitled "Deciphering spatial domains from spatially resolved transcriptomics with Siamese Graph Autoencoder" by Lei Cao, et al. explores an innovative framework called SGAE for spatial domain identification in spatial transcriptomics (ST) data analysis. This framework addresses the limitations of existing methods by incorporating the power of Siamese Graph Autoencoder (SGAE) to improve representation discrimination. The article also highlights the potential of SGAE in enhancing the accuracy of identifying 3D Drosophila embryonic structures. Nevertheless, several significant concerns remain regarding the author's stated conclusion.1ï¼‰ The author mentioned "GNN-based methods suffer from representation collapse, wherein all spatial spots are projected onto a singular representation." and "SGAE mitigates the information correlation at the both sample and feature level, thus improving the representation discrimination." However, the author did not provide enough evidence to demonstrate the ability of SGAE in solving representation collapse, nor did they prove that the GNN approach indeed leads to such problems. More analysis evidence required to support the role of SGAE in representation collapse. Additionally, it would be helpful to understand the impact of representation collapse on downstream bioinformatic analysis.2ï¼‰ Did the Siamese architecture make a difference on the performance of spatial domain identification? Further ablation experiments are needed to demonstrate the effectiveness of each module in the SGAE architecture.3ï¼‰ The author only analyzed the trajectory of one mouse brain sample. This is still far from proving that the embeddings generated by SGAE can exhibit better performance in downstream applications. Does the result of trajectory inference correspond to the actual biological development process?4ï¼‰ Did author adapt proper parameter on the other candidate models? Or is there any different on the preprocess of SGAE and other candidate method? Some of the candidate methods showed significantly poorer results compared to what was reported in corresponding paper, For example the Fig2 A.5ï¼‰ The author mainly used K-means to generate a pseudo-label when pre-clustering. While Louvain or Leiden is used to preform clustering on cell embeddings. Is there any preference of SGAE on choose the pre-cluster method and final cluster method?6ï¼‰ It would be helpful to include a comprehensive hyperparameters table in order to elucidate the relative impact of each parameter on the model's performance. This table should also provide insights into the potential consequences of adjusting these parameters to higher or lower values.7ï¼‰ The author declaimed little batch effect detected on 3D Drosophila datasets. Is there any evidence? How would SGAE performance on multi-slice datasets with high batch effect.

Level of Interest

Please indicate how interesting you found the manuscript: Choose an item.

Quality of Written English

Please indicate the quality of language in the manuscript: Choose an item.

Declaration of Competing Interests

Please complete a declaration of competing interests, considering the following questions:

- Have you in the past five years received reimbursements, fees, funding, or salary from an organisation that may in any way gain or lose financially from the publication of this manuscript, either now or in the future?
- Do you hold any stocks or shares in an organisation that may in any way gain or lose financially from the publication of this manuscript, either now or in the future?
- Do you hold or are you currently applying for any patents relating to the content of the manuscript?
- Have you received reimbursements, fees, funding, or salary from an organization that holds or has applied for patents relating to the content of the manuscript?
- Do you have any other financial competing interests?
- Do you have any non-financial competing interests in relation to this paper?

If you can answer no to all of the above, write 'I declare that I have no competing interests' below. If your reply is yes to any, please give details below.

I declare that I have no competing interests

I agree to the open peer review policy of the journal. I understand that my name will be included on my report to the authors and, if the manuscript is accepted for publication, my named report including any attachments I upload will be posted on the website along with the authors' responses. I agree for my report to be made available under an Open Access Creative Commons CC-BY license (http://creativecommons.org/licenses/by/4.0/). I understand that any comments which I do not wish to be included in my named report can be included as confidential comments to the editors, which will not be published.

Choose an item.

To further support our reviewers, we have joined with Publons, where you can gain additional credit to further highlight your hard work (see: https://publons.com/journal/530/gigascience). On publication of this paper, your review will be automatically added to Publons, you can then choose whether or not to claim your Publons credit. I understand this statement.

Yes Choose an item.
