# Supplementary material for: Deciphering spatial domains from spatially resolved transcriptomics with Siamese graph autoencoder
Source: Gigascience. 2024 Feb 20;13:giae003. doi: 10.1093/gigascience/giae003 (PMC10939418; doi:10.1093/gigascience/giae003)
Supplement: giae003_Reviewer_3_Report_Original_Submission — Ruoyan Li -- 12/14/2023 [file giae003_reviewer_3_report_original_submission.pdf]

Reviewer Report

Title: Deciphering spatial domains from spatially resolved transcriptomics with Siamese Graph Autoencoder

Version: Original Submission **Date:** 12/14/2023

Reviewer name: Ruoyan Li

Reviewer Comments to Author:

In the manuscript entitled 'Deciphering spatial domains from spatially resolved transcriptomics with Siamese Graph Autoencoder', Cao et al developed a new computational framework for spatial domain identification in spatial transcriptomics data. The new framework (SGAE) incorporates the power of Siamese Graph Autoencoder, which mitigates the information correlation at both sample and feature levels. Through a series of benchmarking based on ST datasets generated from different platforms, the authors show that SGAE outperforms other ST clustering methods. Particularly, SGAE has shown its potential for extension and application in multi-slice 3D reconstruction and tissue structure investigation.Overall, the manuscript describes a useful computational framework for ST data clustering and spatial domain identification. The method is sound and the manuscript is well-written.A few points need to be addressed before publication.1. The authors used datasets from different ST platforms and various tissues to examine the performance of SGAE and benchmark against other ST clustering methods. The ST data used in the current manuscript are all from tissues with clear structures such as the mouse cortex and human dorsolateral prefrontal cortex. However, to show the versatility of SGAE, can the authors test its performance when handling more complex and heterogenous tissues like tumors. It would be useful to show the results.2. The authors benchmarked SGAE based on several metrics including ARI, NMI, and FMI. However, the current description of these benchmarking metrics and how exactly benchmarking has been conducted (in the method section) is rudimentary in the manuscript. More detailed information in both the main text and the method section is essential for a better understanding of the method and manuscript.3. More tutorials regarding how to use SGAE need to be included on the Github page.4. The current resolution of all figures in the manuscript is poor, which largely influences the reading. Can authors improve the figure resolution in revision?

Level of Interest

Please indicate how interesting you found the manuscript: Choose an item.

Quality of Written English

Please indicate the quality of language in the manuscript: Choose an item.

Declaration of Competing Interests

Please complete a declaration of competing interests, considering the following questions:

- Have you in the past five years received reimbursements, fees, funding, or salary from an organisation that may in any way gain or lose financially from the publication of this manuscript, either now or in the future?
- Do you hold any stocks or shares in an organisation that may in any way gain or lose financially from the publication of this manuscript, either now or in the future?
- Do you hold or are you currently applying for any patents relating to the content of the manuscript?
- Have you received reimbursements, fees, funding, or salary from an organization that holds or has applied for patents relating to the content of the manuscript?
- Do you have any other financial competing interests?
- Do you have any non-financial competing interests in relation to this paper?

If you can answer no to all of the above, write 'I declare that I have no competing interests' below. If your reply is yes to any, please give details below.

NA

I agree to the open peer review policy of the journal. I understand that my name will be included on my report to the authors and, if the manuscript is accepted for publication, my named report including any attachments I upload will be posted on the website along with the authors' responses. I agree for my report to be made available under an Open Access Creative Commons CC-BY license (http://creativecommons.org/licenses/by/4.0/). I understand that any comments which I do not wish to be included in my named report can be included as confidential comments to the editors, which will not be published.

Choose an item.

To further support our reviewers, we have joined with Publons, where you can gain additional credit to further highlight your hard work (see: https://publons.com/journal/530/gigascience). On publication of this paper, your review will be automatically added to Publons, you can then choose whether or not to claim your Publons credit. I understand this statement.

Yes Choose an item.
